# Age-associated methionine sulfoxide reductase A protects against valvular interstitial cell senescence and valvular calcification

**DOI:** 10.1007/s11357-025-01675-w

**Published:** 2025-05-08

**Authors:** Qing Li, Chengxiang Song, Zisong Wei, Hao Zhou, Shuoding Wang, Hongde Li, Haoran Yang, Qiang Luo, Junli Li, Mao Chen

**Affiliations:** 1 Laboratory of Cardiac Structure and Function, Institute of Cardiovascular Diseases, West China Hospital, Sichuan University, Chengdu, 610041 China; 2 Cardiac Structure and Function Research Key Laboratory of Sichuan Province, West China Hospital, Sichuan University, Chengdu, 610041 China; 3 National Clinical Research Center for Geriatrics, The Center of Gerontology and Geriatrics, West China Hospital, Sichuan University, Chengdu, 610041 China; 4 Department of Cardiology, West China Hospital, Sichuan University, Chengdu, 610041 China; 5 Department of Clinical Research Management, West China Hospital, Sichuan University, Chengdu, 610041 China

**Keywords:** Calcific aortic valve disease, Methionine sulfoxide reductase A, Senescence, Toll-like receptor 2, Nuclear factor-κB

## Abstract

Calcific aortic valve disease (CAVD) is a cardiovascular disease prevalent in the aging population, resulting in high morbidity and mortality rates. However, the molecular mechanisms underlying CAVD remain unclear. We initially conducted an RNA sequencing analysis of aortic valve leaflets from rats of different ages to identify key genes involved in valvular aging and calcification. Bioinformatics analysis demonstrated that methionine sulfoxide reductase A (MSRA) was crucial to valvular calcification and senescence. To further investigate whether and how MSRA influences CAVD pathogenesis, we utilized two in vitro models: a human valvular interstitial cell (VIC) calcification model induced by osteogenic medium, and a VIC senescence model induced by hydrogen peroxide. Western blotting, immunofluorescence, flow cytometry, and alkaline phosphatase staining were conducted to evaluate the changes in calcific nodule formation and senescent markers. In vivo, ApoE^−/−^ mice were treated either a normal chow or a high-cholesterol chow to determine the effects of MSRA overexpression on aortic valve calcification and senescence. MSRA silencing increased the osteogenic differentiation and senescence of VIC, whereas its overexpression produced the opposite effects. Similarly, we found that MSRA overexpression reduced calcium deposition and decreased the levels of senescent markers in ApoE^−/−^ mice. Further mechanism experiments showed that MSRA suppressed osteoblastic differentiation via inhibiting the toll-like receptor (TLR2)/nuclear factor-κB (NF-κB) pathway. Our findings demonstrate that MSRA ameliorates valvular calcification and senescence by inhibiting TLR2/NF-κB pathway, highlighting MSRA as a promising target for treating age-associated CAVD.

**Supplementary Information:**

The online version contains supplementary material available at 10.1007/s11357-025-01675-w.

## Introduction

Calcific aortic valve disease (CAVD), a complex cardiovascular disorder associated with aging, is becoming increasingly prevalent and is associated with substantial morbidity and mortality in the elderly population [1, 2]. To date, there are no available pharmaceutical therapies for CAVD other than transcatheter or surgical aortic valve replacement [3]. Moreover, the intraoperative complications and the long-term postoperative outcomes remain unsatisfactory, particularly in older patients [4]. Hence, comprehensive insights into the molecular mechanisms underlying CAVD may facilitate the development of novel therapeutic approaches for this disease.

CAVD is a chronic pathological process characterized by valvular endothelial injury, inflammatory infiltration, and accumulation of extracellular matrix, ultimately resulting in valvular fibrosis and calcification [5]. Notably, emerging evidence indicates that valvular interstitial cells (VICs), the predominant cell type in all layers of the aortic valve leaflet, can differentiate into osteoblast-like phenotypes in response to various stimuli, a process that plays a critical role in valvular calcification [6, 7]. Additionally, senescent VICs exhibit an increased propensity to acquire osteoblast-like phenotypes [8, 9], suggesting that cellular senescence plays a pivotal role in disease progression.

Cellular senescence is characterized by a state of stable and irreversible cell cycle arrest, induced by diverse stressors, such as aging, DNA damage, and telomere dysfunction [10, 11]. During aging, the expression of genes involved in inflammation, mitochondria, and collagen may change [12, 13], ultimately leading to the release of pro-inflammatory cytokines, accumulation of reactive oxygen species (ROS), and extracellular matrix remodeling, which in turn drives cellular senescence [10]. Senescent cells can also secret biologically active substances, such as matrix metalloproteinase, pro-inflammatory cytokines, and β-galactosidase, which can influence the cellular microenvironment and exacerbate valvular calcification [5]. Thus, targeting age-associated genes may be an effective strategy for identifying potential therapeutic regimens for age-associated CAVD.

Methionine sulfoxide reductase A (MSRA), an antioxidant enzyme that converts methionine sulfoxide to methionine, plays a critical role in maintaining redox homeostasis and extending lifespan [14–16]. Its disruption can result in oxidative modification of biomolecules and cell damage owing to the cumulative effects of ROS, which, in turn, contribute to aging and age-related diseases [17, 18]. Furthermore, previous studies have demonstrated that MSRA is associated with a range of age-related diseases, such as Alzheimer’s disease [19], diabetes mellitus [20], and atherosclerosis diseases [21, 22], which share similar etiologies with CAVD. Given MSRA’s critical role in aging, understanding its function in inhibiting VIC senescence and calcification may render it a novel therapeutic target for treating age-related CAVD.

In this study, we investigated the role of MSRA in the progression of valvular calcification and senescence. We demonstrated that MSRA inhibited osteogenic medium-induced VIC calcification and hydrogen peroxide (H_2_O_2_)-induced VIC senescence. We also found that MSRA overexpression ameliorated aortic valvular calcification in a well-established mouse model of hypercholesterolemia-induced CAVD. Mechanistically, MSRA suppressed VIC calcification by inhibiting toll-like receptor (TLR2)/nuclear factor-κB (NF-κB) pathway. Our findings indicated that MSRA is a novel therapeutic target for the pharmacological treatment of CAVD.

## Materials and methods

### Human aortic valve tissues

Calcific aortic valve leaflets were acquired from individuals with CAVD who underwent aortic valve replacement. Non-calcific leaflets were acquired from individuals with aortic dissection, aortic valve prolapse, or aortic sinus dilatation who underwent surgery. Non-calcific tissues were further detected by gross observation and microscopic analysis of hematoxylin and eosin (H&E)-stained cryosections to ensure the absence of calcific nodules. The baseline characteristics of individuals are presented in Supplementary Table 1. This study adhered to the principles of the Declaration of Helsinki and received approval from the Ethics Committee of the West China Hospital of Sichuan University (2017 Trial Number 93). All participants provided written informed consent before participating in the study.

### Human VIC culture and treatment

Primary human VICs were isolated from non-calcific aortic valve leaflets, digested with collagenase II (17,101,015, Gibco, Thermo Fisher Scientific, Waltham, USA), and cultured in Dulbecco’s modified Eagle’s medium (DMEM) (C11995500BT, Gibco, Thermo Fisher Scientific, Waltham, USA) supplemented with 10% fetal bovine serum (BC-SE-FBS01, Bio-Channel, Nanjing, China) and 1% penicillin/streptomycin (15,140–122, Gibco, Thermo Fisher Scientific, Waltham, USA) under conditions of 37 °C and 5% CO2 atmosphere as previously described [23, 24]. The cells were used for subsequent experiments at passages 2–5.

To induce VIC calcification, cells were cultured in osteogenic medium containing DMEM, 5% fetal bovine serum, 1% penicillin/streptomycin, 10 mmol/L β-glycerophosphate (154,804–51-0, Sigma-Aldrich, St. Louis, USA), 50 μg/mL ascorbic acid (49,752, Sigma-Aldrich, St. Louis, USA), and 100 nmol/L dexamethasone (HY-14648, MCE, NJ, USA) [23]. The osteogenic medium was replaced every 3–4 days and the VICs were collected at designated time points on days 3, 5, and 10. To induce VIC senescence, 50 μM hydrogen peroxide (H_2_O_2_) was added into medium for 3 days and changed with fresh medium without H_2_O_2_ for another 2 days [25].

To further explore the role of MSRA in calcification or senescence, gain- and loss-of-function experiments were performed prior to the induction of calcification or senescence. To knockdown MSRA, VICs with 70 to 80% confluent in plates were incubated with a mixture of MSRA siRNA (50 nM) (sense: 5′- GGGACAGACUUUCUACUAUTT-3′; antisense: 5′-AUAGUAGAAAGUCUGUCCCTT-3′) (GenePharma, Shanghai, China), Lipofectamine™ RNAiMAX (13,778,150, Invitrogen, Carlsbad, USA), and Opti-MEM (31,985,070, Thermo Fisher Scientific, Waltham, USA) for 8 h, and then cell stimulation was performed. Control cells were treated with scrambled siRNA and transfection reagent. To overexpression MSRA, VICs at 70 to 80% confluence in plates were transduced with adenovirus containing MSRA expression (HANBIO, Shanghai, China) at a multiplicity of infection of 50 for 8 h, and then calcification or senescence stimulation was performed. Control cells were transduced with adenoviruses containing empty plasmids.

To determine the role of TLR2/NF-κB in the effect of MSRA on VICs, the TLR2 agonist Pam3CSK4 (0.1 μg/mL, 112,208–01–2, InvivoGen, San Diego, USA) [26, 27] or phosphate buffered saline (PBS) as control was added to osteogenic medium in VICs cultured with and without the MSRA overexpression.

### Animal experiments

To investigate age-associated changes in the mRNA profile of aortic valve tissue, we analyzed gene expression in young (3-month-old), adult (6-month-old), and old (24-month-old) male Sprague–Dawley rats (Beijing Vital River Laboratory Animal Technology Co., Ltd, China). Three biological replicate rats were included in each age group for RNA sequencing. Following euthanasia, hearts were harvested and washed with ice-cold PBS. Using a stereomicroscope, the heart was carefully lifted with forceps, and the left ventricular outflow tract was carefully dissected along the aortic root. The aortic valve leaflets were then exposed and excised by dissection around the annulus. Harvested valves were preserved in TRIzol reagent (15596026 CN, Invitrogen, CA, USA). Total RNA was immediately extracted and purified using an RNAeasy Kit (Qiagen). Library preparation and transcriptome sequencing were performed using an Illumina NovaSeq6000 platform (Beijing, China). Mapping of 150-bp paired-end reads to genes was generated using HTSeq v0.6.0 software, and fragments per kilobase of transcript per million fragments mapped were calculated. Finally, we analyzed the RNA-sequencing data and found that the differentially expressed gene (DEG) expression that gradually increased or decreased with age, with a *p*-value threshold of < 0.05. Next, the DEGs from rats were intersected with genes associated with aging/longevity from Human Ageing Genomic Resources (https://genomics.senescence.info/). Subsequently, 18 aging-related genes overlapped with CAVD-associated genes identified from the public gene expression profile series GSE51472 (https://www.ncbi.nlm.nih.gov/geo/query/acc.cgi?acc=GSE51472), which included data on the gene expression profiles of five normal human aortic valves and five calcified human aortic valves. Four candidate genes were identified as potential regulators of CAVD.

To investigate the role of MSRA in a well-established mouse model of hypercholesterolemia-induced CAVD, 6-week-old male ApoE^−/−^ mice with C57BL/6 background were sourced from Beijing Vital River Laboratory Animal Technology Co., Ltd (China). Adeno-associated virus subtype 2 vectors overexpression MSRA (AAV2-MSRA) and controls (AAV2-control) were purchased from Genechem Co., Ltd (Shanghai, China). Before high-cholesterol diet (HCD) treatment (H10141, HFK, Beijing, China), AAV2 (8.0 × 10^11^ vector genomes/per mouse) was injected through the tail vein as previously described [24]. The ApoE^−/−^ mice were randomly divided into four groups: (1) normal diet (ND) + AAV2-control (*n* = 9); (2) HCD + AAV2-control (*n* = 10); (3) ND + AAV2-MSRA (*n* = 9); and (4) HCD + AAV2-MSRA (*n* = 10). ApoE^−/−^ mice were observed for 24 weeks to assess valvular calcification [24, 28]. The echocardiographic parameters of all the mice were examined via transthoracic echocardiography using a 15–70-MHZ array probe (MX500D) connected to a Vevo 3100 imaging system (Fujifilm Visualsonics, Toronto, Canada). All animals were sacrificed, and aortic valves and blood samples were obtained for subsequent histopathological examinations. Serum glucose, total cholesterol (TC), triglyceride (TG), low-density lipoprotein cholesterol (LDL-C), and high-density lipoprotein cholesterol (HDL-C) levels were examined using an automated biochemical analyzer (Cobas 8000 C702, Roche, Basel, Switzerland) following the manufacturer’s instructions.

All animals were kept in a pathogen-free environment at 23 ± 2 °C with 50–60% humidity under a 12-h light/dark cycle, with free access to chow and water. All animal experiments were approved by the Animal Care and Use Committee of the West China Hospital of Sichuan University (20,220,920,001), and all procedures were executed in accordance with the European Communities Council Directives 86/609/EEC and 2010/63/EU.

### RNA extraction and quantitative real-time PCR

RNA was extracted from treated VICs or tissues using TRIzol reagent, and 500 ng of RNA was reverse transcribed into cDNA using the HiScript III RT SuperMix (R323, Vazyme, Nanjing, China), following the manufacturer’s instructions. Quantitative real-time polymerase chain reaction (PCR) was carried out using ChamQ SYBR Color qPCR Master Mix (Q411, Vazyme, Nanjing, China) and an ABI QuantStudio 7 Flex sequence detection system (Thermo Fisher Scientific, Waltham, USA). The primers used for real-time PCR are listed in Supplementary Table 2. Glyceraldehyde-3-phosphate dehydrogenase (GAPDH) served as the internal control for normalization, and relative quantification was calculated using the 2^−ΔΔCt^ method.

### Western blot analysis

Protein was extracted from human VICs using radio immunoprecipitation assay lysis buffer (89,900, Thermo Fisher Scientific, Waltham, USA) containing protease/phosphatase inhibitors (78,440, Thermo Fisher Scientific, Waltham, USA). VIC lysate samples were then separated on 10% or 12.5% sodium dodecyl sulfate–polyacrylamide gel electrophoresis (SDS-PAGE) gels (PG112/PG113, EpizymeBiotech, Shanghai, China) and transferred to 0.22-μm polyvinylidene difluoride (PVDF) membranes (ISEQ00010, Merck Millipore, Billerica, USA). The PVDF membranes were treated with 5% bovine serum albumin (BSA) (4240GR100, BioFroxx, Guangzhou, China) for 1 h and subsequently incubated with primary antibodies overnight at 4 °C. Primary antibodies against osteocalcin (OCN) (A20800, Abclonal, Wuhan, China, dilution 1:1000), runt-related transcription factor 2 (RUNX2) (A11753, Abclonal, Wuhan, China, dilution 1:1000), osteopontin (OPN) (ab214050, Abcam, Cambridge, USA, dilution 1:1000), cyclin-dependent kinase inhibitor 1 A (P21) (10,355–1-AP, Proteintech, Wuhan, China, dilution 1:1000), MSRA (14,547–1-AP, Proteintech, Wuhan, China, dilution 1:1000), TLR2 (66,645–1-Ig, Proteintech, Wuhan, China, dilution 1:1000), phosphorylated NF-κB p65 (3033, CST, Danvers, USA, dilution 1:1000), NF-κB p65 (8242, CST, Danvers, USA, dilution 1:1000), and GAPDH (A19056, Abclonal, Wuhan, China) were used. Membranes were then incubated with anti-rabbit or anti-mouse horseradish peroxidase (HRP)-conjugated secondary antibodies (7074/7076, CST, Danvers, USA, dilution 1:1000) for 1 h at room temperature. Thereafter, the protein-antibody complex was examined using an enhanced chemiluminescence assay (P10060, NCM Biotech, Suzhou, China). Finally, band intensity was measured using Fiji ImageJ software (version 2.14.0, Bethesda, USA), and relative protein expression was calculated as the ratio of the target protein intensity to that of GAPDH.

### Alkaline phosphatase staining

VIC calcification was determined by alkaline phosphatase (ALP) staining (C3206, Beyotime, Shanghai, China). After 10 days of osteogenic medium induction, VICs were fixed in 4% paraformaldehyde (PFA) solution (60536ES76, YEASEN, Shanghai, China) for 30 min at room temperature, followed by incubation with freshly prepared ALP solution overnight. Subsequently, the samples were photographed immediately (Sony α6000, Tokyo, Japan). The ALP-positive cell area was visualized as a dark blue-violet color, and Fiji ImageJ software was employed to quantify the ratio of the positively stained cell area to the total cell area.

### SA-β-galactosidase staining

SA-β-galactosidase (SA-β-gal) staining kit (C0602, Beyotime, Shanghai, China) was used to stain senescent cells. Briefly, cells were fixed in 4% PFA solution for 30 min at room temperature and then treated with freshly prepared β-gal solution at 37 °C overnight. Blue-stained VICs were regarded as senescent VICs, and Fiji ImageJ software was used to quantify the ratio of the positively stained cell area to the total cell area.

### Measurement of ROS levels

After treatment, an ROS assay kit (S0033S, Beyotime, Shanghai, China) was used to measure the intracellular ROS levels. Briefly, VICs were washed twice with serum-free DMEM medium and then treated with 10 mM DCFH-DA for 20 min at 37 °C in the dark, followed by three washes in serum-free DMEM medium. Finally, the VICs were digested with trypsin, and the mean fluorescence intensity was calculated using flow cytometry (BD LSRFortessa, Franklin Lakes, USA).

### Annexin V-FITC/PI staining

An Annexin V-FITC/PI apoptosis detection kit (A211, Vazyme, Nanjing, China) was applied to detect apoptotic VICs. Briefly, VICs were digested with trypsin in each well. Afterwards, cells were stained with freshly mixed Annexin V-FITC/PI staining solution for 20 min in the dark at room temperature. The samples were immediately detected using flow cytometry. Annexin V +/PI − cells were identified as apoptotic cells.

### Immunofluorescence staining

For immunofluorescence staining, VICs were washed thrice with PBS and fixed with 4% PFA solution for 10 min. VICs were permeabilized using 0.1% Triton-X (20107ES76, YEASEN, Shanghai, China) for 5 min and then blocked with 5% BSA for 60 min. Afterwards, cells were treated with primary antibodies overnight at 4 °C. The primary antibodies were as follows: MSRA (14,547–1-AP, Proteintech, Wuhan, China, dilution 1:200), RUNX2 (12,556, CST, Danvers, USA, dilution 1:1000), P21 (10,355–1-AP, Proteintech, Wuhan, China, dilution 1:200), TLR2 (66,645–1-Ig, Proteintech, Wuhan, China, dilution 1:200), and NF-κB p65 (6956, CST, Danvers, USA, dilution 1:200). Next day, VICs were incubated with the Alexa Fluor 488–labeled secondary antibody (A11034, Thermo Fisher Scientific, Waltham, dilution 1:500) or Alexa Fluor 555–labeled secondary antibody (A32727, Thermo Fisher Scientific, Waltham, dilution 1:500) for 60 min at 37 °C. Finally, the VICs were mounted using anti-fade reagents with 4′,6-diamidino-2-phenylindole (DAPI) (S36938, Invitrogen, Carlsbad, USA). Imaging was performed using a confocal laser scanning microscope (STELLARIS 5, Leica, Wetzlar, Germany).

### Flow cytometry

Briefly, VICs were digested with trypsin in each treatment well. After fixation with 4% PFA and permeabilization with 0.5% Triton-X, VICs were stained with primary antibodies against MSRA (PA5-14,206, Thermo Fisher Scientific, Waltham, USA, dilution 1:50), RUNX2 (dilution 1:50), or P21 (dilution 1:50) for 30 min. Afterwards, VICs were treated with Alexa Fluor 488–labeled secondary antibody in the dark for 30 min at 37 ℃. Finally, the mean immunofluorescence intensity was detected via flow cytometry (BD LSRFortessa, BD Biosciences, Franklin Lakes, USA).

### Enzyme-linked immunosorbent assay of cytokines

The pro-inflammatory cytokine levels, including tumor necrosis factor-α (TNF-α), monocyte chemoattractant protein-1(MCP-1), interleukin-1β (IL-1β), and IL-6, were measured in mouse serum samples using the enzyme linked immunosorbent assay kit (RK04328, ABclonal, Wuhan, China) in accordance with the manufacturer’s guidelines.

### H&E and Von Kossa staining

Briefly, aortic valve leaflet samples from human and mice were fixed in 4% PFA, embedded in paraffin, and sectioned at 5-µm thickness for further experiments. H&E staining followed a standard protocol, and the thickness of the aortic valve leaflets was measured as previously described [24]. Von Kossa staining (G1043, Servicebio, Wuhan, China) was used to evaluate calcific nodules in the aortic valve leaflets. Additional tissue sections were treated with 5% silver nitrate and exposed to ultraviolet light for 60 min following the manufacturer’s protocols. Subsequently, the samples were stained with sodium thiosulfate for 2 min and stained with eosin. Calcification was measured as the ratio of the calcific area to the aortic valve leaflets using the average optical density method in Fiji ImageJ software [29].

### Immunohistochemistry

The 5-µm paraffin-embedded aortic valve tissues were used for immunohistochemistry staining. Briefly, after antigen retrieval, tissues were treated with primary antibodies overnight, followed by staining with secondary antibodies for 60 min. The primary antibodies used were as follows: MSRA (ab16803, Abcam, Cambridge, USA, dilution 1:1000 for human samples or 1:500 for mouse samples) and P21 (10,355–1-AP, Proteintech, Wuhan, China, dilution 1:1800 for human samples, or 1:1000 for mouse samples). The slides were then incubated in diaminobenzidine (DAB4033, MXB, Fujian, China) solution for 2 min and counterstained with hematoxylin for 5 min. Images were captured using a Leica DMi8 microscope (Wetzlar, Germany) and analyzed using the average optical density method in Fiji ImageJ software.

### Proteomics analysis

Proteomics was conducted to compare protein expression profiles in VICs treated with siRNAMSRA versus siRNAscramble under osteogenic medium conditions. In brief, treated VICs in three biological replicates were collected and were sent to Applied Protein Technology (Shanghai, China) for 4D-label-free liquid chromatography–tandem mass spectrometry analysis. The raw protein data for each sample were quantitatively analyzed using MaxQuant 1.6.14 software. The results were analyzed to identify differentially expressed proteins and to perform Kyoto Encyclopedia of Genes and Genomes (KEGG) pathway analysis.

### Statistical analysis

Continuous data were reported as means ± standard error of the mean, whereas categorical data were presented as number (percentage). All the results were obtained from at least three independent experiments. For comparisons between two groups, Student’s *t*-test or Mann–Whitney *U* test was used, whereas one-way analysis of variance followed by Bonferroni post hoc test or Kruskal–Wallis test followed by Dunn’s post hoc test was applied for multiple group comparisons. Categorical data were compared using the chi-square test. Statistical significance was considered as a two-sided *p*-value < 0.05, with adjustments for multiple comparisons made using Bonferroni correction or Dunn’s post hoc test, depending on the context. All analyses were performed using GraphPad Prism (version 8.2.1, GraphPad Software Inc., La Jolla, CA, USA) and SPSS software (version 25.0, IBM Corp., Armonk, USA).

## Results

### Identification of key genes during senescence and calcification of aortic valve

**Fig. 1:**
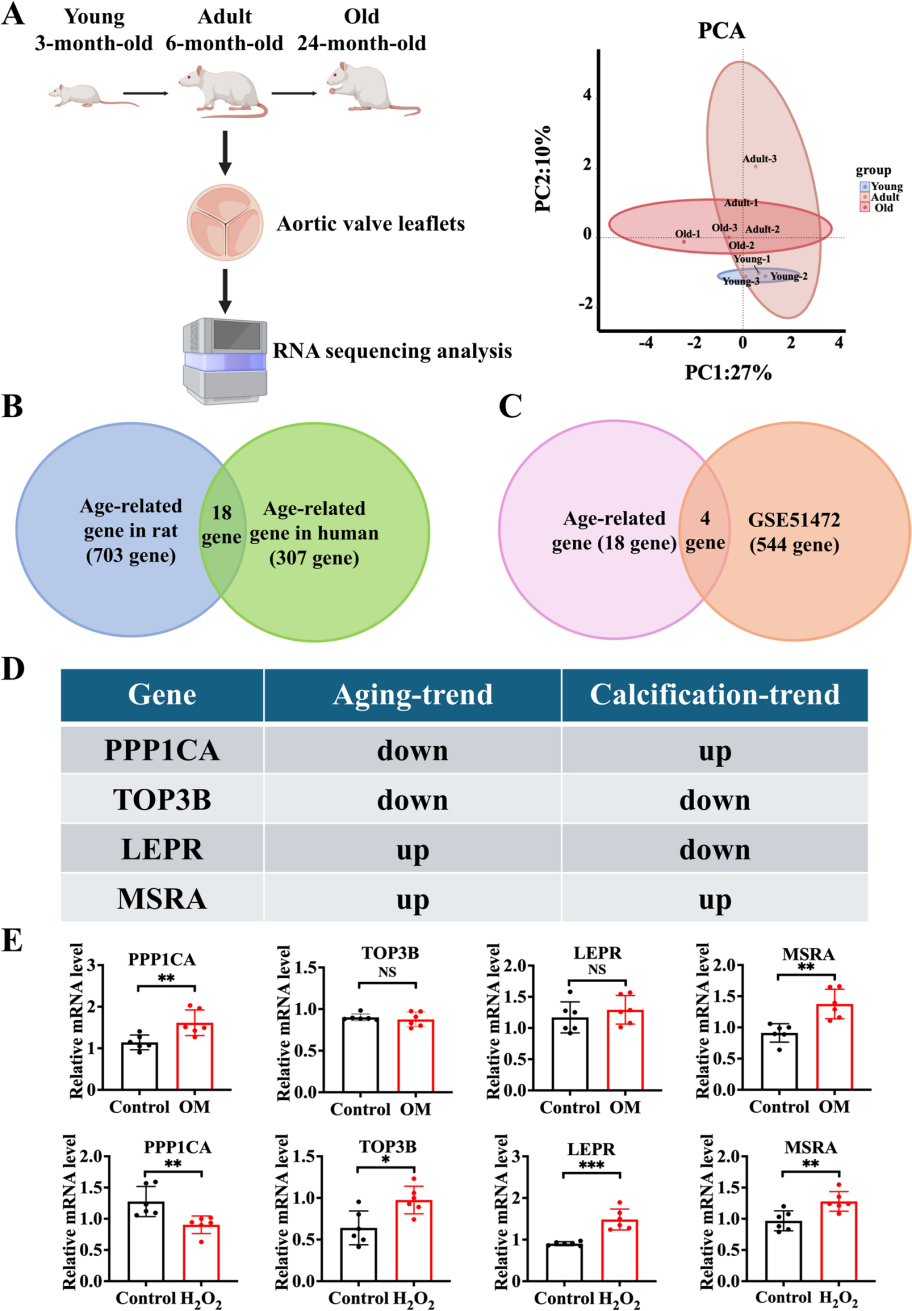
Identification of key genes during age-associated CAVD. **A** RNA sequencing analysis of aortic valve leaflets from male Sprague–Dawley rats of different ages (3-month-old, 6-month-old, and 24-month-old) (*n* = 3 for each group). Venn diagram showing the overlapped differentially expressed genes of **B** aging and **C** age-associated CAVD (GSE51472 included gene expression data for CAVD patients and controls and was downloaded from the GEO database). **D** The potential key genes related to age-associated CAVD. **E** Quantitative real-time polymerase chain reaction analysis of mRNA levels of key genes in OM-induced calcified VIC model and H_2_O_2_-induced senescent VIC model (*n* = 6 for each group). Data are presented as mean ± SEM and compared by Student’s *t*-test. CAVD, calcific aortic valve disease; VICs, valvular interstitial cells; OM, osteogenic medium; H_2_O_2_, hydrogen peroxide; PPP1CA, protein phosphatase 1 catalytic subunit α; TOP3B, topoisomerase 3β; LEPR, leptin receptor; MSRA, methionine sulfoxide reductase A. NS, not significant; *P < 0.05, **P < 0.01, ***P < 0.001

To delineate the dynamic changes in gene expression profiles during aortic valvular aging, we analyzed RNA sequencing data from the aortic valve leaflets of Sprague–Dawley rats of different ages (Fig. 1A). Our analysis identified 703 DEGs that gradually increased or decreased in expression levels with age in aortic valve tissues. Subsequently, we identified 18 overlapping DEGs that persisted in the human aging/longevity genes (Fig. 1B). Given that CAVD is a degenerative disease associated with aging, we further intersected these age-related DEGs with CAVD-associated genes from the public gene expression dataset GSE51472. Four candidate genes were identified as potential genes associated with CAVD in subsequent analyses (Fig. 1C). Notably, the expression of topoisomerase 3β (TOP3B) and MSRA was consistent across both aging and calcification process (Fig. 1D). However, only MSRA expression was consistently validated in the aging and calcified VICs (Fig. 1E). Thus, MSRA was the focus of our subsequent experiments.

### *MSRA protects against VIC osteoblastic differentiation *in vitro

**Fig. 2:**
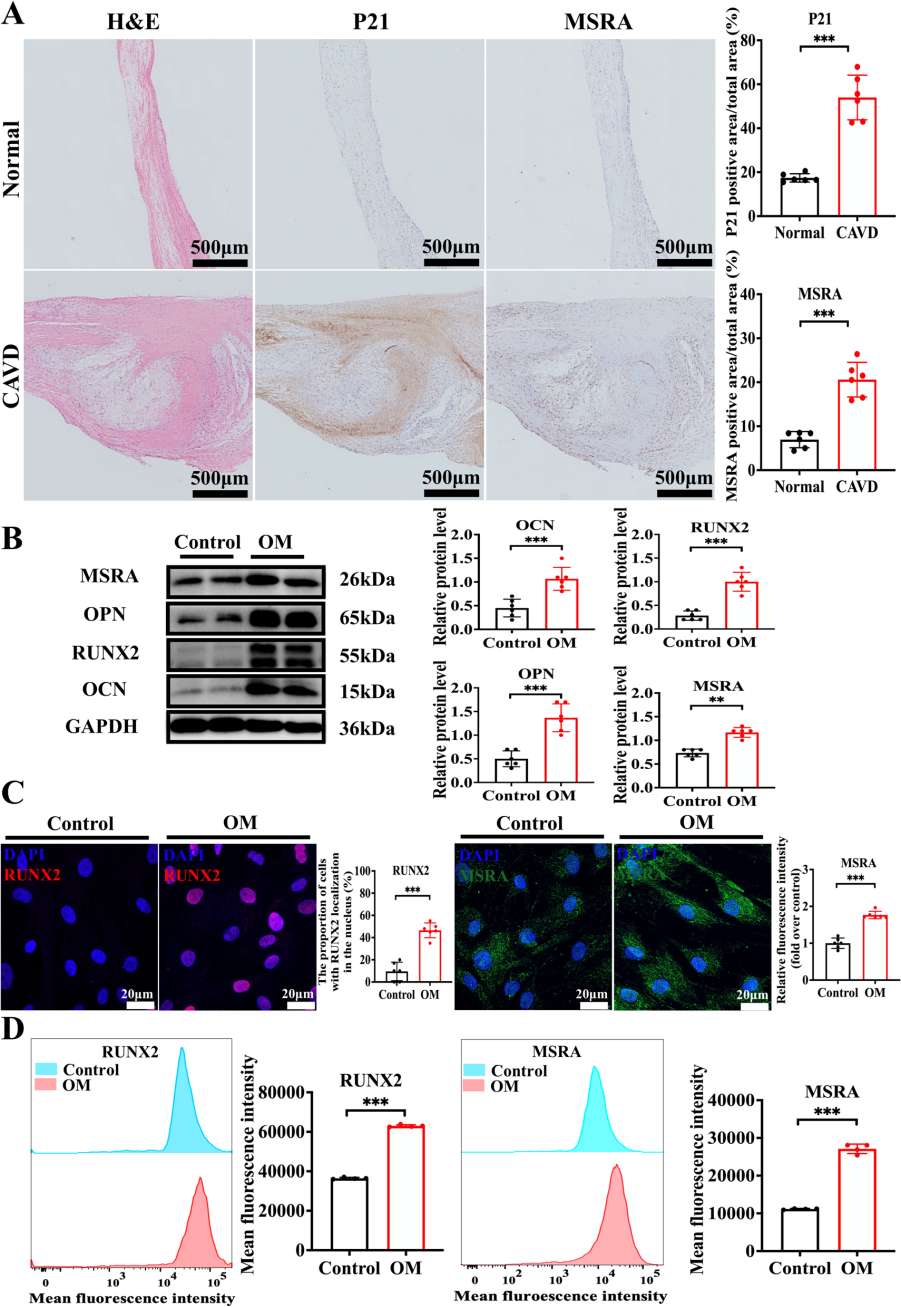
MSRA expression is upregulated in CAVD. **A** H&E staining showing the normal and calcific human aortic valve tissues and immunohistochemical staining showing P21 and MSRA expression in the normal and calcific human aortic valve tissues (*n* = 6 for each group), scale bar = 500 μm. **B** Western blot analysis and quantification of OCN, RUNX2, OPN, and MSRA protein expression in calcified VICs and controls (*n* = 6 for each group). **C** Immunofluorescence staining and quantification of RUNX2 (red) and MSRA (green) in calcified VICs and controls (*n* = 6 for each group), scale bar = 20 μm. **D** Flow cytometry analysis and the mean fluorescence intensity of RUNX2 and MSRA in calcified VICs and controls (*n* = 4 for each group). Data are presented as mean ± SEM and compared by Student’s *t*-test. MSRA, methionine sulfoxide reductase A; CAVD, calcific aortic valve disease; H&E, hematoxylin-and eosin; OM, osteogenic medium; OCN, osteocalcin; RUNX2, runt-related transcription factor; OPN, osteopontin; VICs, valvular interstitial cells; DAPI, 4′,6-diamidino-2-phenylindole. NS, not significant; *P < 0.05, **P < 0.01, ***P < 0.001

**Fig. 3:**
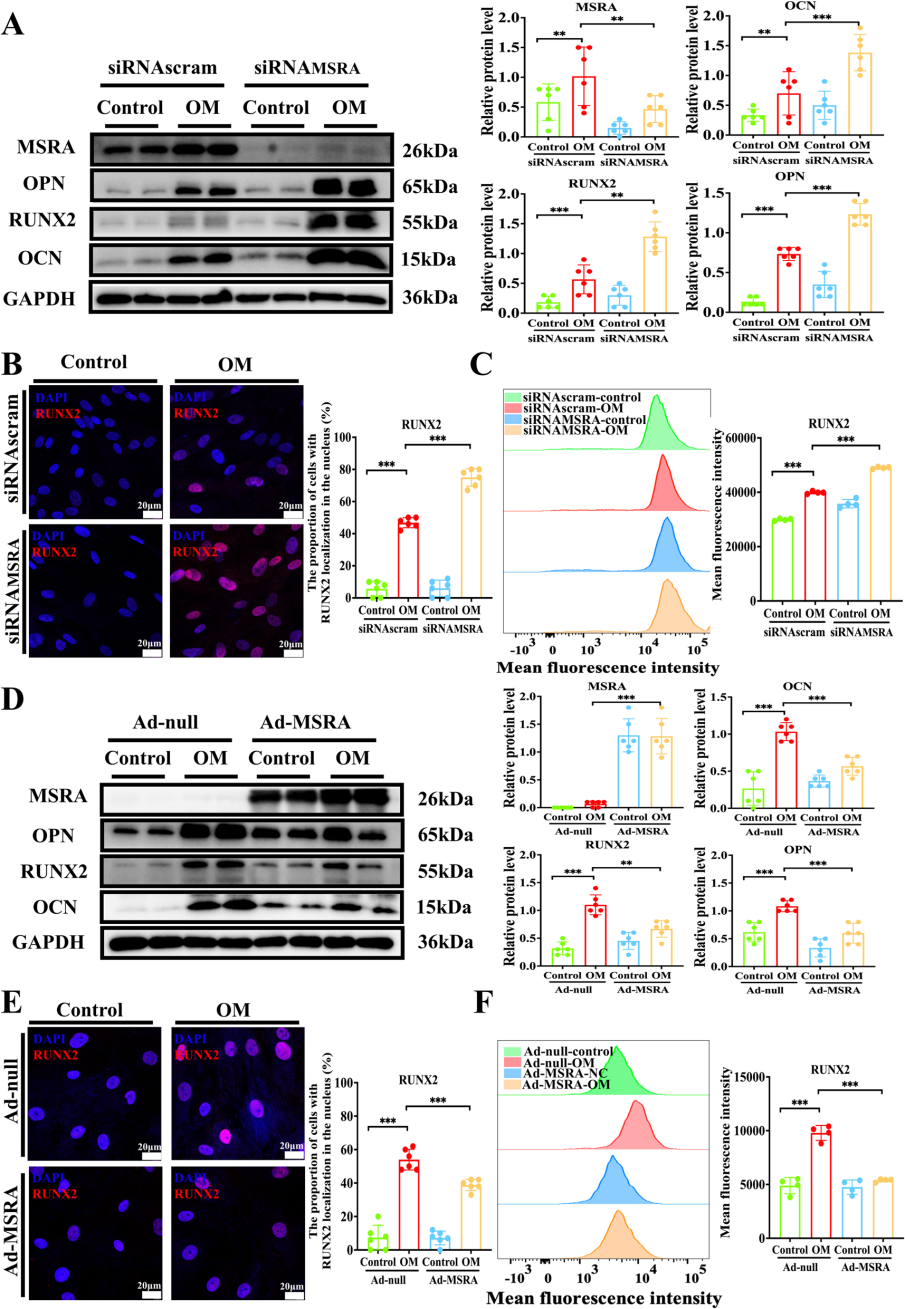
MSRA alleviates osteogenic differentiation of human VICs in vitro. **A** Western blot analysis and quantification of OCN, RUNX2, OPN, and MSRA protein expression in VICs under osteogenic medium or control medium following MSRA silencing (*n* = 6 for each group). **B** Immunofluorescence staining and quantification of RUNX2 (red) under osteogenic medium or control medium after MSRA silencing (*n* = 6 for each group), scale bar = 20 μm. **C** Flow cytometry analysis and the mean fluorescence intensity of RUNX2 in VICs under osteogenic medium or control medium after MSRA silencing (*n* = 4 for each group). **D** Western blot analysis and quantification of OCN, RUNX2, OPN, and MSRA protein expression in VICs under osteogenic medium or control medium following MSRA overexpression (*n* = 6 for each group). **E** Immunofluorescence staining and quantification of RUNX2 (red) under osteogenic medium or control medium after MSRA overexpression (*n* = 6 for each group), scale bar = 20 μm. **F** Flow cytometry analysis and the mean fluorescence intensity of RUNX2 in VICs under osteogenic medium or control medium after MSRA overexpression (*n* = 4 for each group). Data are presented as means ± SEM and compared by Student’s *t*-test or one-way analysis of variance followed by Bonferroni post hoc test. MSRA, methionine sulfoxide reductase A; OM, osteogenic medium; OCN, osteocalcin; RUNX2, runt-related transcription factor; OPN, osteopontin; VICs, valvular interstitial cells; DAPI, 4′,6-diamidino-2-phenylindole. NS, not significant; *P < 0.05, **P < 0.01, ***P < 0.001

To validate these observations, we examined the role of the MSRA in valvular calcification. We initially examined the MSRA expression in normal and calcific human aortic valve leaflets. Immunohistochemistry results demonstrated elevated MSRA expression in calcific aortic valve leaflets compared with that in normal aortic valve leaflets (Fig. 2A). We also evaluated its expression in VICs under osteogenic medium induction and found that MSRA protein expression was increased in calcific VICs, accompanied by elevated levels of osteogenic markers, such as OCN, RUNX2, and OPN (Fig. 2B–D). To further explore the function of MSRA in CAVD, gain- and loss-of-function experiments were carried out using a VIC calcification model in vitro. MSRA knockdown via siRNA and overexpression of adenoviruses were performed. Silencing of MSRA expression significantly increased the expression of osteogenic markers, including OCN, RUNX2, and OPN (Fig. 3A–C), as well as calcific nodule formation in VICs cultured under osteogenic conditions (Supplementary Fig. 1). Additionally, we observed that MSRA depletion increased ROS production and apoptotic cell formation in VICs cultured in osteogenic medium (Supplementary Fig. 2 A–B). In contrast, adenovirus-mediated MSRA overexpression produced a significantly decline in the levels of calcific markers (Fig. 3D–E) and calcium deposition (Supplementary Fig. 3). MSRA overexpression also reduced the formation of ROS and apoptotic cells in VICs cultured in osteogenic medium (Supplementary Fig. 4 A–B). Collectively, these data support that MSRA protects against the osteogenic differentiation of VICs and ROS formation.

### *MSRA protects against VIC senescence *in vitro

**Fig. 4:**
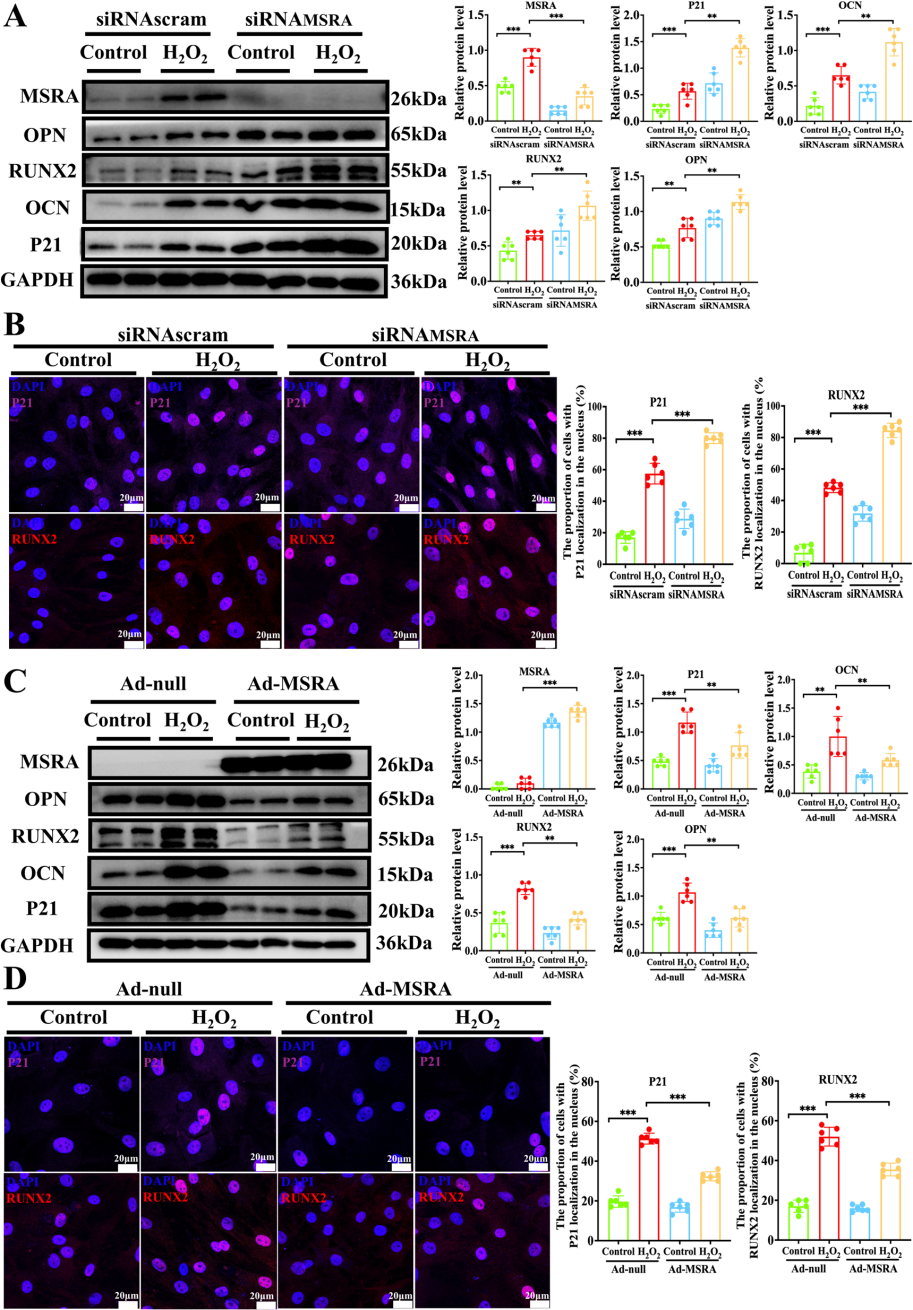
MSRA alleviates VIC senescence in vitro. **A** Western blot analysis and quantification of P21, OCN, RUNX2, OPN, and MSRA protein expression in VICs under H_2_O_2_ stimulus after MSRA silencing (*n* = 6 for each group). **B** Immunofluorescence staining and quantification of P21 (magenta) and RUNX2 (red) in VICs under H_2_O_2_ stimulus after MSRA silencing (*n* = 6 for each group), scale bar = 20 μm. **C** Western blot analysis and quantification of P21, OCN, RUNX2, OPN, and MSRA protein expression in VICs under H_2_O_2_ stimulus after MSRA overexpression (*n* = 6 for each group). **D** Immunofluorescence staining and quantification of P21 (magenta) and RUNX2 (red) in VICs under H_2_O_2_ stimulus after MSRA overexpression (*n* = 6 for each group), scale bar = 20 μm. Data are presented as means ± SEM and compared by Student’s *t*-test or one-way analysis of variance followed by Bonferroni post hoc test. MSRA, methionine sulfoxide reductase A; P21, cyclin-dependent kinase inhibitor 1 A; H_2_O_2_, hydrogen peroxide; OCN, osteocalcin; RUNX2, runt-related transcription factor; OPN, osteopontin; VICs, valvular interstitial cells; DAPI, 4′,6-diamidino-2-phenylindole. NS, not significant; *P < 0.05, **P < 0.01, ***P < 0.001

Senescence has been reported to contribute to valvular calcification [8], which is consistent with our results that senescent marker P21 was upregulated in calcified aortic valve leaflets (Fig. 2A). Therefore, we used an H_2_O_2_-induced cellular senescence model to study the function of senescent VICs in the calcification process. Senescent marker P21 and osteogenic markers were upregulated upon H_2_O_2_ stimulation (Supplementary Fig. 5 A–C). We further observed that the senescent stimulus H_2_O_2_ exacerbated osteogenic medium-induced VIC calcification, suggesting that senescent VICs were prone to acquiring an osteogenic phenotype (Supplementary Fig. 6). Next, we found that MSRA expression was upregulated in senescent VICs (Supplementary Fig. 5 A–C). We then knocked down MSRA in VICs and applied a senescent stimulus to further explore its function. As expected, MSRA knockdown increased VIC senescence and was more likely to acquire an osteogenic phenotype (Fig. 4A–B and Supplementary Fig. 7). In contrast, MSRA overexpression inhibited VIC senescence and was less likely to acquire an osteogenic phenotype (Fig. 4C–D and Supplementary Fig. 8). Taken together, these findings indicate that MSRA protects against VIC senescence during valvular calcification.

### MSRA suppresses osteoblastic differentiation by inhibiting TLR2/NF-κB pathway

**Fig. 5:**
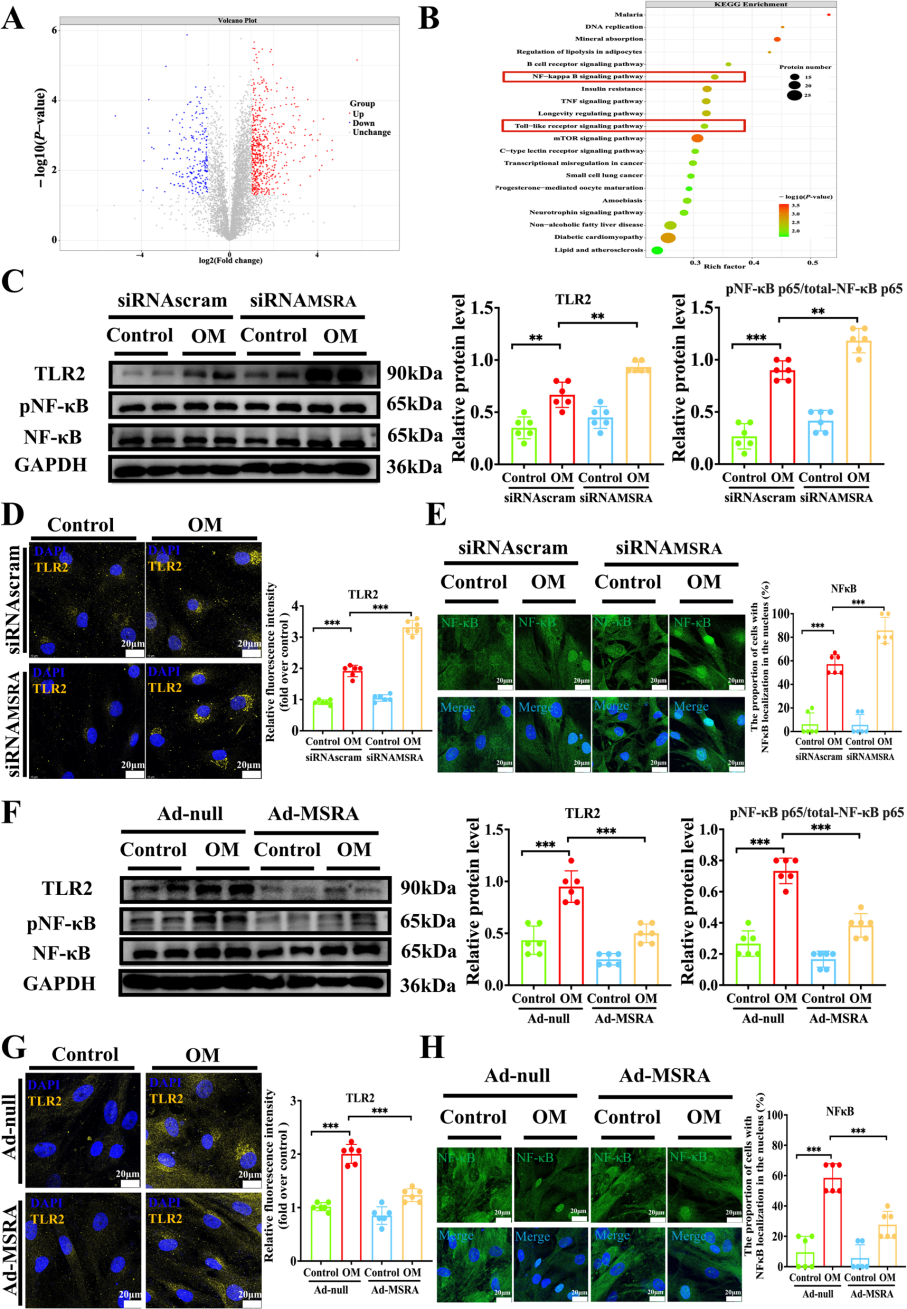
MSRA inhibits the TLR2/NF-κB pathway during the osteogenic differentiation of VICs. **A** Volcano plot representing differentially expressed proteins in VICs treated with siRNAMSRA versus siRNAscramble under osteogenic medium conditions. Differentially expressed proteins were screened upon the threshold of adjusted *P*-value < 0.05 and |log2 (fold change)|≥ 1. Upregulated proteins are presented in red dots and downregulated proteins are presented in blue dots (*n* = 3 for each group). **B** The bubble plot illustrating KEGG pathway enrichment analysis of differentially expressed proteins between depleted MSRA VICs and control VICs under osteogenic medium induction (*n* = 3 for each group). The *Y*-axis represents the top 20 different KEGG terms, the *X*-axis represents protein rich factor enriched in relative KEGG terms, the circle size refers to protein numbers, and the color represents *P*-value. **C** Western blot analysis and quantification of TLR2, pNF-κB p65/total NF-κB p65 expression following MSRA silencing (*n* = 6 for each group). **D** Immunofluorescence staining and quantification of TLR2 (yellow) in VICs during the calcification process following MSRA silencing, scale bar = 20 μm. **E** Immunofluorescence staining and quantification of NF-κB (green) following MSRA silencing (*n* = 6 for each group), scale bar = 20 μm. **F** Western blot analysis and quantification of TLR2, pNF-κB p65/total NF-κB p65 expression following MSRA overexpression (*n* = 6 for each group). **G** Immunofluorescence staining and quantification of TLR2 (yellow) in VICs during the calcification process following MSRA overexpression (*n* = 6 for each group), scale bar = 20 μm. **H** Immunofluorescence staining and quantification of NF-κB (green) following MSRA overexpression (*n* = 6 for each group), scale bar = 20 μm. Data are presented as means ± SEM and compared by Student’s *t*-test or one-way analysis of variance followed by Bonferroni post hoc test. MSRA, methionine sulfoxide reductase A; TLR2, toll-like receptor 2; pNF-κB, phosphorylated nuclear factor-κB; KEGG, Kyoto Encyclopedia of Genes and Genomes; VICs, valvular interstitial cells; OM, osteogenic medium; DAPI, 4′,6-diamidino-2-phenylindole. NS, not significant; *P < 0.05, **P < 0.01, ***P < 0.001

**Fig. 6:**
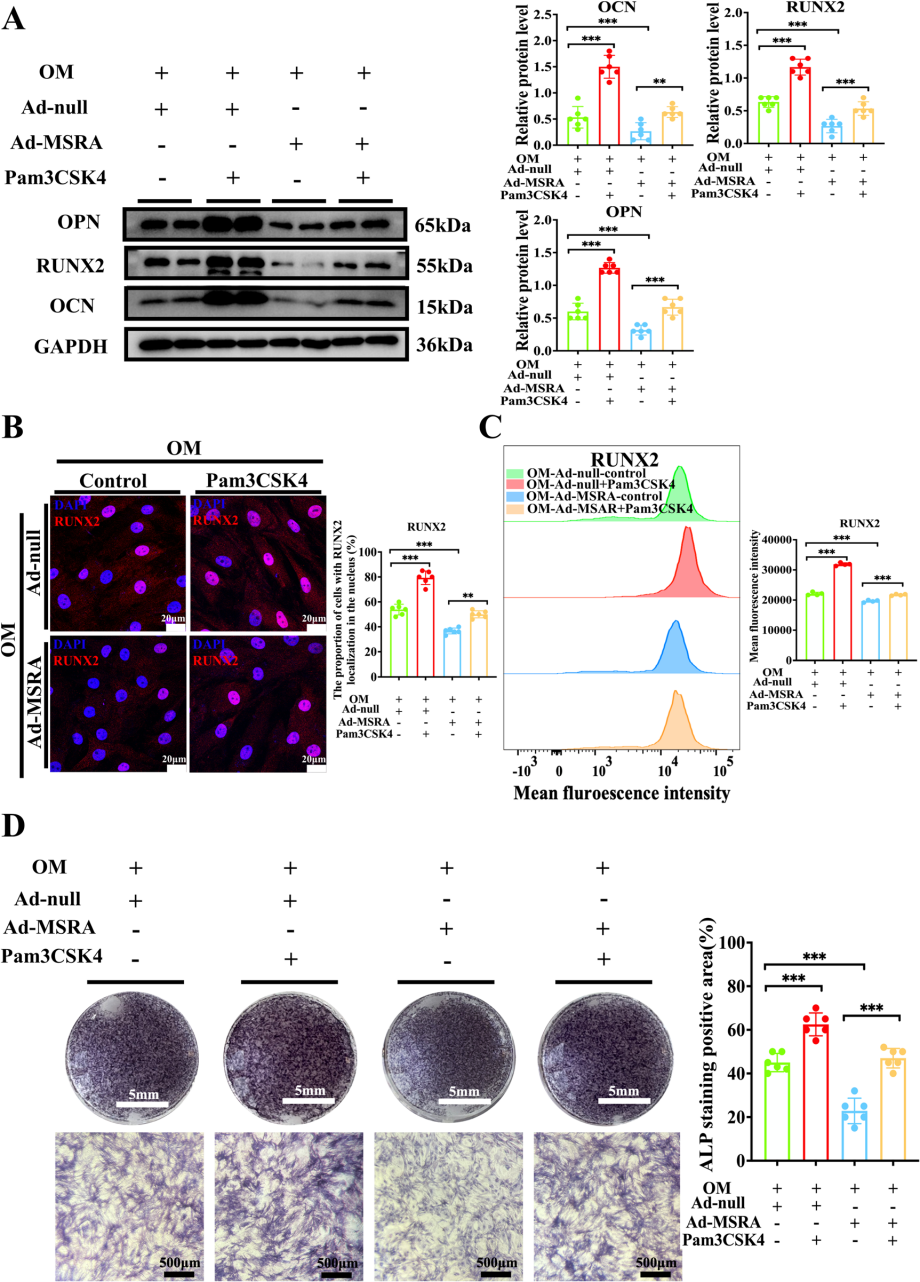
MSRA alleviates osteogenic differentiation of VICs by inhibiting the TLR2/NF-κB pathway. **A** Western blot analysis and quantification of OCN, RUNX2, and OPN protein expression when VICs were cultured in osteogenic medium with or without TLR2 agonist Pam3CSK4 (0.1 μg/mL) after MSRA overexpression (*n* = 6 for each group). **B** Immunofluorescence staining and quantification of RUNX2 (red) when human VICs were cultured in osteogenic medium with or without TLR2 agonist Pam3CSK4 after MSRA overexpression (*n* = 6 for each group), scale bar = 20 μm. **C** Flow cytometry analysis and the mean fluorescence intensity of RUNX2 when human VICs were cultured in osteogenic medium with or without TLR2 agonist Pam3CSK4 after MSRA overexpression (*n* = 4 for each group). **D** Alkaline phosphatase staining when VICs were cultured in osteogenic medium with or without TLR2 agonist Pam3CSK4 after MSRA overexpression (*n* = 6 for each group), scale bar = 5 mm and 500 μm. Data are presented as means ± SEM and compared by Student’s *t*-test or one-way analysis of variance followed by Bonferroni post hoc test. MSRA, methionine sulfoxide reductase A; VICs, valvular interstitial cells; OM, osteogenic medium; OCN, osteocalcin; RUNX2, runt-related transcription factor; OPN, osteopontin; DAPI, 4′,6-diamidino-2-phenylindole. NS, not significant; *P < 0.05, **P < 0.01, ***P < 0.001

To explore the mechanism of the impact of MSRA on VIC calcification, we carried out proteomics profiling in VICs with depleted MSRA expression using siRNA under osteogenic medium induction. Proteomics analysis displayed 796 differentially expressed proteins, of which 573 were significantly upregulated and 223 were downregulated. KEGG analysis of the differentially expressed proteins indicated that MSRA silencing regulates multiple biological processes, including TLR and NF-κB signaling pathway (Fig. 5A and 5B). However, the TLRs that are activated during MSRA silencing in CAVD are unknown. Therefore, we examined the TLR2, TLR3, and TLR4 mRNA levels after MSRA silencing and found that only TLR2 expression was significantly increased (Supplementary Fig. 9). We observed that silencing MSRA expression in VICs increased the protein expression of TLR2 and phosphorylated NF-κB p65/total NF-κB p65 (Fig. 5C–D). Besides, MSRA depletion significantly increased the nuclear translocation of NF-κB p65 in VICs cultured in osteogenic medium (Fig. 5E). Conversely, adenovirus-mediated overexpression of MSRA in VICs significantly reduced the protein expression of TLR2 and phosphorylated NF-κB p65/total NF-κB p65 (Fig. 5F–G). Additionally, overexpression of MSRA in VICs reduced the nuclear translocation of NF-κB (Fig. 5H). To confirm whether MSRA exerts its functions through inhibiting the TLR2/NF-κB pathway, we performed a rescue experiment. We used Pam3CSK4 (a TLR2 agonist) to treat VICs in the presence of osteogenic medium, together with MSRA overexpression. Pam3CSK4 promoted the expression of osteogenic markers, including OCN, RUNX2, and OPN (Fig. 6A–C) and calcium deposition (Fig. 6D), but this effect was abrogated by MSRA overexpression (Fig. 6A–D). Collectively, these results strongly suggest that MSRA protects against osteogenic differentiation by inhibiting TLR2/NF-κB pathway in VICs.

### *MSRA overexpression alleviates aortic valve calcification and senescence in ApoE*^*−/−*^* mice*

**Fig. 7:**
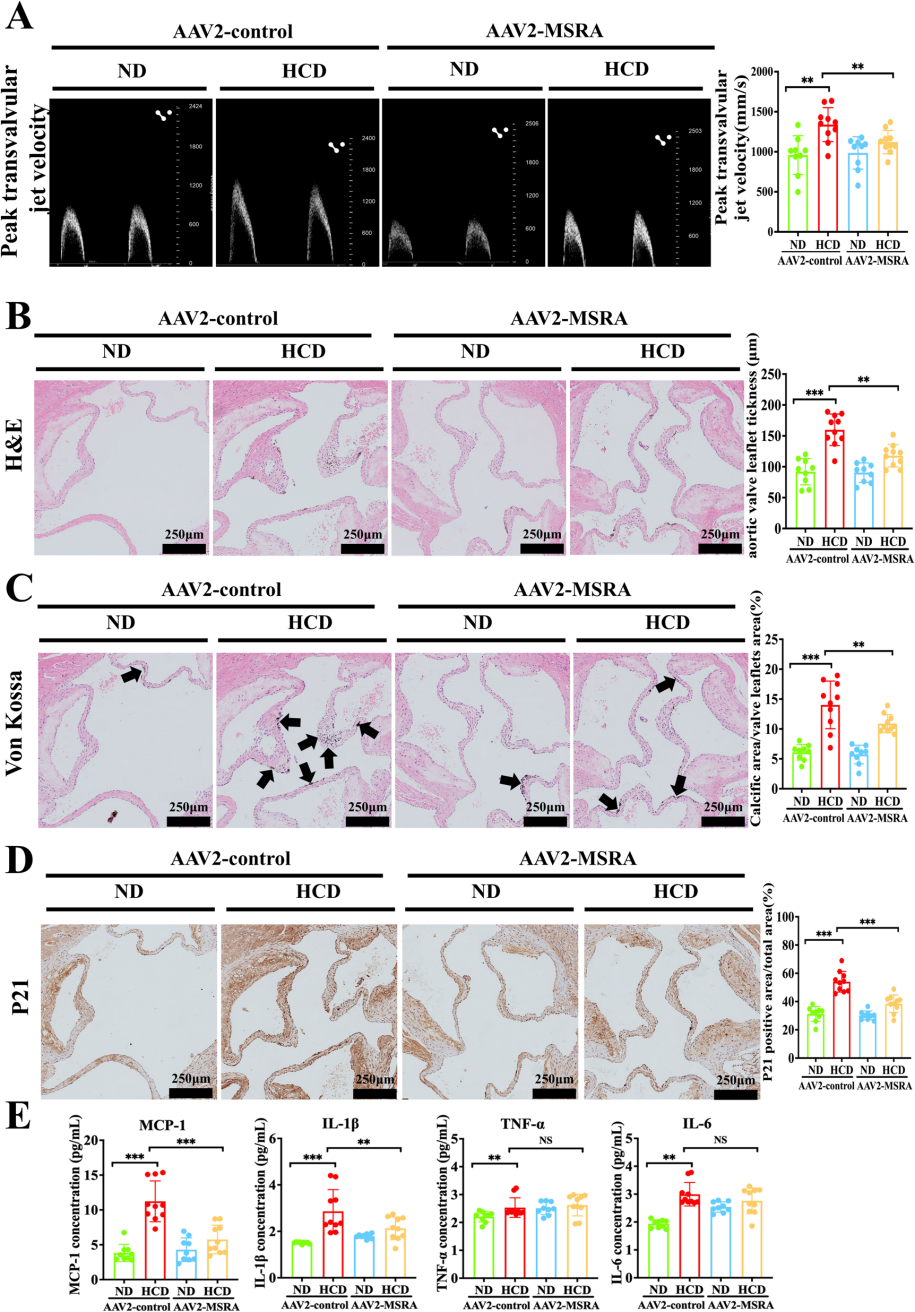
MSRA overexpression alleviates aortic valve calcification and senescence in vivo.** A** Echocardiographic data peak transvalvular jet velocity in ApoE^−/−^ mice (*n* = 9 for each ND group, *n* = 10 for each HCD group). **B** H&E staining of the aortic valve leaflets in ApoE^−/−^ mice. **C** Von Kossa staining of the aortic valve leaflets in ApoE^−/−^ mice (*n* = 9 for each ND group, *n* = 10 for each HCD group), scale bar = 250 μm. **D** Immunohistochemical staining showed P21 in ApoE^−/−^ mice (*n* = 9 for each ND group, *n* = 10 for each HCD group), scale bar = 250 μm. **E** The inflammatory cytokines in ApoE.^−/−^ mice (*n* = 9 for each ND group, *n* = 10 for each HCD group). Data are presented as means ± SEM and compared by Student’s *t*-test or one-way analysis of variance followed by Bonferroni post hoc test. MSRA, methionine sulfoxide reductase A; H&E, hematoxylin-and eosin; AAV2, adeno-associated virus subtype 2; ND, normal diet; HCD, high cholesterol diet; P21, cyclin-dependent kinase inhibitor 1 A; MCP-1, monocyte chemoattractant protein-1; IL, interleukin; TNF, tumor necrosis factor. NS, not significant; *P < 0.05, **P < 0.01, ***P < 0.001

To assess the role of MSRA in valvular calcification and senescence in vivo, we overexpressed MSRA in ApoE^−/−^ mice by administering AAV2-MSRA (or AAV2-control) (Supplementary Fig. 10). After 24 weeks, ApoE^−/−^ mice in the HCD + AAV2-control group exhibited significant augments in both peak transvalvular jet velocity (Fig. 7A) and aortic valve leaflet thickness (Fig. 7B) compared to those in the ND + AAV2-control group. MSRA overexpression partly restored the change. Moreover, Von Kossa staining showed HCD-accelerated calcification, as evidenced by increased calcium deposition in the aortic valve leaflets of ApoE^−/−^ mice (Fig. 7C). Strikingly, MSRA overexpression partially normalized this change. Similarly, we observed that the senescent marker P21 was reduced in the aortic valve leaflets of ApoE^−/−^ mice overexpressing MSRA (Fig. 7D). Moreover, MSRA overexpression significantly reduced pro-inflammatory cytokines MCP-1 and IL-1β levels, but this effect was not detected in TNF-α and IL-6 levels (Fig. 7E). MSRA overexpression also reduced total cholesterol, triglyceride, and low-density lipoprotein cholesterol levels, but failed to modulate glucose and high-density lipoprotein cholesterol levels (Supplementary Table 3). These findings demonstrate that MSRA overexpression markedly alleviates aortic valve calcification and senescence in ApoE^−/−^ mice.

## Discussion

CAVD is a cardiovascular disease with high morbidity and mortality in the aging population. It is caused by the osteogenic differentiation of VICs and increased inflammation, with no drug therapies currently available for its treatment. In the present study, we found that the age-associated gene, MSRA, ameliorated calcification and senescence in VICs. In HCD-fed ApoE^−/−^ mice, a well-recognized experimental CAVD model, MSRA overexpression suppressed CAVD development and progression. Our mechanistic studies displayed that MSRA alleviated VIC conversion to osteoblast-like cells via downregulation of the TLR2/NF-κB pathway. Our research elucidates the functional role and the underlying mechanisms of MSRA in protecting against CAVD.

The primary finding of our research was that MSRA protected against the osteogenic differentiation of VICs and the progression of CAVD. MSRA, a key endogenous antioxidant enzyme, reduces the oxidation of methionine sulfoxide back to methionine, thereby mitigating oxidative damage and ultimately extending lifespan [18]. Beyond its role in aging, the lack of MSRA has been linked to metabolic dysregulation, as evidenced by its deficiency exacerbating insulin resistance in high-fat diet-fed mice, suggesting its involvement in glucose homeostasis [20]. Moreover, MSRA-knockout mice exhibit heightened susceptibility to angiotensin II-induced cardiac apoptosis and increased mortality following myocardial infarction compared with their wild-type counterparts [30]. Conversely, MSRA overexpression has been shown to significantly attenuate atherosclerosis by reducing lipid and pro-inflammatory cytokine levels in western-diet-fed mice [22]. Additionally, targeted enhancement of MSRA expression via a cell-penetrating peptide also ameliorates atherosclerosis in ApoE^−/−^ mice by bolstering its antioxidative and anti-inflammatory capacity [21]. Despite these established cardiovascular benefits, the role of MSRA in valvular calcification remains largely unexplored. Given the shared pathophysiological mechanisms of cardiovascular diseases and CAVD, we hypothesized that MSRA exerts a similar protective effect against aortic valvular disease. To address this, we performed both gain- and loss-of-function experiments, which demonstrated that MSRA attenuated the osteogenic differentiation of VICs in vitro. Furthermore, MSRA overexpression significantly mitigated high-fat diet-induced aortic valvular calcification in an in vivo animal model. Collectively, these findings suggest that MSRA is a potential therapeutic target for preventing the progression of valvular calcification.

Another important finding of our research is that MSRA serves as a protective agent against VIC senescence. As a key regulator of redox balance, MSRA reduces oxidized methionine residues, thereby preventing oxidative damage to proteins and maintaining mitochondrial function, both of which are crucial for delaying cellular senescence [31]. MSRA plays a protective role in cardiomyocytes by reducing their susceptibility to oxidative stress, and its deficiency has been linked to increased mitochondrial impairment and cardiac dysfunction [32]. Additionally, MSRA can prevent the oxidation of methionine residues in IκBα, thereby inhibiting NF-κB activation and mitigating inflammation, which is a major contributor to cardiovascular aging [33]. Furthermore, MSRA directly influences cell cycle progression by modulating P53 acetylation, which in turn influences the transcription of the cyclin-dependent kinase inhibitor P21, a key mediator of senescence [34]. Given its role in mitigating oxidative stress and regulating key senescence-associated pathways, MSRA has emerged as a critical factor in maintaining cardiovascular homeostasis. Our findings extend this understanding by highlighting its protective function in valvular aging and paving the way for future studies exploring its therapeutic potential in age-related valvular dysfunction.

Interestingly, our study found that MSRA expression was upregulated during aging, which contradicts with previous reports indicating an age-related decline in its expression [35, 36]. We hypothesized that this upregulation represents an endogenous compensatory mechanism aimed at mitigating oxidative damage in senescent cells. Similar compensatory responses have been observed in other antioxidant systems under stress conditions [37, 38]. Previous studies have primarily focused on MSRA expression in organs such as the liver, kidney, and brain, whereas its expression in cardiac tissues has been less extensively investigated, and even less so in heart valves. To date, one study has explored MSRA expression in cardiac tissue and reported that both MSRA mRNA and protein levels are elevated in aged hearts [39]. This suggests that the regulation of MSRA expression in aging cardiac tissues differs from that observed in other organs. Moreover, we observed that its upregulation occurred during the late stages of senescence (Supplementary Fig. 11 A and 11B). At this point, ROS had already accumulated (Supplementary Fig. 11 C), and both cellular senescence and calcification were well-established. Thus, the increase in MSRA expression at this point was inadequate to counteract age-related damage. These findings suggest that early intervention to enhance MSRA expression may be more effective in preventing the onset of valvular senescence and calcification.

Another interesting of our finding is that MSRA modulated inflammation by regulating the TLR2/NF-κB pathway. Accumulating evidence indicates that MSRA downregulates the activation of the inflammatory pathway through its antioxidative repair function [22, 40]. The anti-inflammatory role of MSRA is particularly relevant in atherosclerosis diseases [21, 22]. Given that the inflammation pathway plays a critical role in the pathogenesis of CAVD and accelerates the aging process, particularly through the NF-κB-mediated pathway, understanding the regulatory mechanisms of MSRA is crucial [10, 41]. NF-κB is a well-known signal pathway that transduces signals from TLRs and subsequently modulates the expression of pro-inflammatory cytokines [42]. However, it remains unexplored whether or which TLR is responsible for NF-κB activation during MSRA silencing in age-associated CAVD. To the best of our knowledge, this is the first research to demonstrate that TLR2, rather than TLR3 or TLR4, participates in calcification following MSRA silencing in human VICs. Furthermore, we observed that the TLR2 agonist Pam3 CSK4 abolished the protective effect of MSRA overexpression by inhibiting the osteogenic differentiation of VICs. Together, these data suggest that MSRA overexpression suppresses osteogenic response of VICs and calcific nodules by inhibiting the TLR2/NF-κB pathway. However, the precise molecular mechanisms underlying this regulatory process remain unclear. Future work is warranted to further elucidate the downstream signaling cascades and explore the therapeutic potential of targeting MSRA in age-related CAVD.

Targeting MSRA expression is a promising therapeutic strategy for mitigating valvular calcification and cellular senescence. One potential approach is dietary intervention, as methionine restriction, particularly in early life, has been shown to enhance MSRA expression through activation of the FOXO signaling pathway, contributing to an extended lifespan [43]. Additionally, small-molecule activators such as resveratrol and S-methyl-L-cysteine can upregulate MSRA and reduce oxidative damage in cardiovascular disease models [44, 45]. Beyond dietary and pharmacological strategies, protein-based approaches have also been explored. For example, Wu et al. developed a PEP-1-MSRA fusion protein to enhance intracellular MSRA delivery, significantly mitigating atherosclerosis in a western-diet-fed ApoE^−/−^ mouse model [21]. Despite these promising preclinical findings, the translation of MSRA-targeted therapies into clinical applications remains challenging owing to species-specific metabolic differences, tissue-specific gene regulation, and potential off-target effects. Further research is necessary to optimize these therapeutic strategies, refine delivery methods, and evaluate their long-term safety and efficacy in human. Exploring innovative delivery systems such as gene therapy and nanoparticle-based approaches may further enhance the clinical potential of MSRA modulation in age-related CAVD.

## Limitations

Our research had several limitations. First, human primary VICs cultured in vitro may have altered properties when compared to VICs in vivo. However, we confirmed the function of MSRA using ApoE^−/−^ mice in vivo. Second, we only observed that MSRA suppressed the osteoblastic differentiation of VICs by inhibiting the TLR2/NF-κB p65 signaling pathway. Given that signaling pathways often interact or crosstalk in cellular responses, we cannot rule out the possibility that MSRA suppresses the osteogenic responses of VICs through additional pathways. Third, the precise mechanisms underlying the protective role of MSRA against valvular calcification and senescence were not explored in this study. Fourth, although H₂O₂ does not fully replicate the complexity of human aging, it is a widely accepted agent for inducing oxidative stress-induced senescence in vitro [25, 46]. Fifth, while Pam3CSK4 is recognized for its potent agonistic effect on TLR2, which enhances the activation of receptors and subsequent inflammatory response pathways [27], it can also activate TLR1, indicating its ability to simultaneously influence other TLR pathways [47]. Finally, although the high-fat diet-fed ApoE^−/−^ mouse is well-established for studying CAVD, its pathophysiological features do not entirely mirror those of human disease. Future work is warranted to elucidate the underlying molecular mechanisms.

## Conclusions

In summary, our findings show that age-associated MSRA inhibits the osteoblastic transformation of VICs by inhibiting TLR2/NF-κB p65-mediated inflammation. Our findings may lead to a paradigm shift in pharmacological therapy for CAVD and identify potential targets for CAVD management strategies.

## Supplementary Information

Below is the link to the electronic supplementary material.

Supplementary file1 (DOCX 27.2 MB)

## Data Availability

The data support the findings of this study are available from the corresponding author upon reasonable request.
