# Supplementary material for: Age-associated methionine sulfoxide reductase A protects against valvular interstitial cell senescence and valvular calcification
Source: GeroScience. 2025 May 8;48(1):807–27. doi: 10.1007/s11357-025-01675-w (PMC12972393; doi:10.1007/s11357-025-01675-w)
Supplement: Supplementary file 1 — Supplementary file1 (DOCX 27.2 MB) [file 11357_2025_1675_MOESM1_ESM.docx]

**Supplementary table 1. Clinical characteristics of enrolled participants.**

| Variables | Normal  (n=6) | CAVD  (n=6) | *P*-value |
| --- | --- | --- | --- |
| Age (years) | 51±3.8 | 58±4.9 | <0.001 |
| Male | 6(100%) | 6(100%) | - |
| Current smoker | 3(50%) | 3(50%) | - |
| BMI (kg/m^2^) | 24.8±3.1 | 24.9±4.2 | 0.426 |
| Hypertension | 3(50%) | 3(50%) | - |
| Diabetes mellitus | 2(33.3%) | 2(33.3%) | - |
| TG (mmol/L) | 1.3±0.4 | 1.5±0.4 | 0.248 |
| TC (mmol/L) | 4.2±0.8 | 4.2±0.9 | 0.457 |
| LDL-C (mmol/L) | 2.3±0.6 | 2.4±0.7 | 0.208 |
| HDL-C (mmol/L) | 1.0±0.2 | 1.0±0.3 | 0.458 |
| Mean transvalvular gradient (mmHg) | 23±3.2 | 42±4.8 | <0.001 |
| LVEF (%) | 63±4.4 | 65±5.0 | 0.125 |

Data are expressed as the mean value ± standard deviation or number (%). CAVD, calcific aortic valve disease; BMI, body mass index; TG, triglyceride; TC, total cholesterol; LDL-C, low-density lipoprotein cholesterol; HDL-C, high-density lipoprotein cholesterol; LVEF, left ventricular ejection fraction.

**Supplementary table 2. Primers sequences of quantitative real-time polymerase chain reaction.**

| Gene | Forward sequence (5'-3') | Reverse sequence (5'-3') |
| --- | --- | --- |
| GAPDH (human) | CAAGGCTGTGGGCAAGGTCATC | GTGTCGCTGTTGAAGTCAGAGGAG |
| PPP1CA (human) | TTTCCCTCCCGAGAGCAACTACC | CCGCATGATCCGCCGAATCTG |
| TOP3B (human) | CTGTGGAGATGCTGCGTGTGG | GCTGACCGTGGCGATGAAGTG |
| LEPR (human) | ATGTGTCCTTCCTGATTCTGTGGTG | TGACAACTGTGTAGGCTGGATTGC |
| MSRA (human) | AGAATCACGACCCGACCCAAGG | AGGTACTGCTGGTGGTAGTCTTCC |
| TLR2 (human) | CTACCAGATGCCTCCCTCTTACCC | GTGAGCAGGATCAGCAGGAACAG |
| TLR3 (human) | TTCACCATTCCAGCCTCTTCGTAAC | CCTCAACTGGGATCTCGTCAAAGC |
| TLR4 (human) | TCTCCAACAGCATTTAACTCACTCTCC | CATTCCATTCGTTCAACTTCCACCAAG |
| GAPDH (mouse) | CCTCGTCCCGTAGACAAAATG | TGAGGTCAATGAAGGGGTCGT |
| MSRA (mouse) | TGTATTTGGAATGGGCTGCTTCTGG | TCTCGGATGTCGGTGGTGATGG |

GAPDH, glyceraldehyde-3-phosphate dehydrogenase; PPP1CA, protein phosphatase 1 catalytic subunit α; TOP3B, topoisomerase 3β; LEPR, leptin receptor; MSRA, methionine sulfoxide reductase A; TLR, toll-like receptor.

**Supplementary table 3. Metabolic parameters in different groups of mice.**

| Parameters | ND+AAV2-control  (n=9) | HCD+AAV2-control  (n=10) | ND+AAV2-MSRA  (n=9) | HCD+AAV2-MSRA  (n=10) |
| --- | --- | --- | --- | --- |
| Glucose (mg/dL) | 107.4±7.6 | 97.9±9.5 | 108.7±7.2 | 105.2±6.4 |
| TC (mg/dL) | 577.2±19.7 | 1604±21.2 | 526.0±17.57 | 539.4±14.38 |
| TG (mg/dL) | 122.9±6.3 | 159.8±6.9 | 92.8±8.1 | 94.4±9.7 |
| LDL-C (mmol/L) | 281.7±13.8 | 1358.0±15.7 | 280.5±9.8 | 278.1±13.7 |
| HDL-C(mmol/L) | 80.8±5.5 | 92.7±4.7 | 83.1±4.8 | 83.3±4.8 |

Values are means ± standard error of the mean (SEM); AAV, adeno-associated virus serotype 2; ND, normal diet; HCD, high cholesterol diet; MSRA, methionine sulfoxide reductase A; TC, total cholesterol; TG, triglyceride; LDL-C, low-density lipoprotein cholesterol; HDL-C, high-density lipoprotein cholesterol.

**Supplementary figure 1.** **Alkaline phosphatase staining for calcium deposition in VICs after MSRA silencing (n=6 for each group), scale bar: 5mm and 500μm.** Data are presented as means ± SEM and compared by one-way analysis of variance followed by Bonferroni post-hoc test. VICs, valvular interstitial cells; MSRA, methionine sulfoxide reductase A; OM, osteogenic medium.


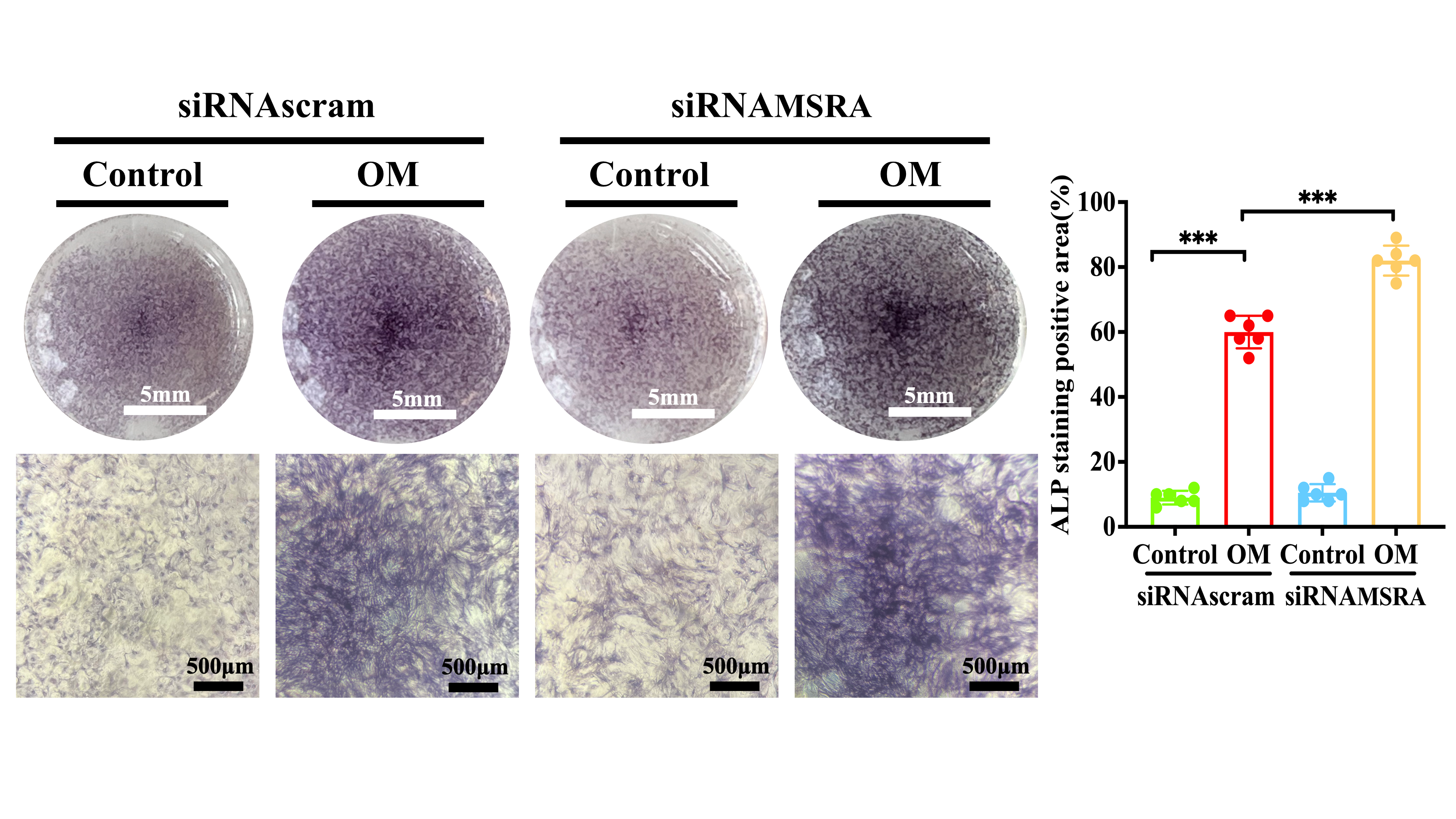


**Supplementary figure 2.** **MSRA silencing increases (A) ROS production and (B) apoptotic cell formation in VICs culture with osteogenic medium (n=4 for each group).** Data are presented as means ± SEM and compared by one-way analysis of variance followed by Bonferroni post-hoc test. MSRA, methionine sulfoxide reductase A; ROS, reactive oxygen species; VICs, valvular interstitial cells; OM, osteogenic medium.


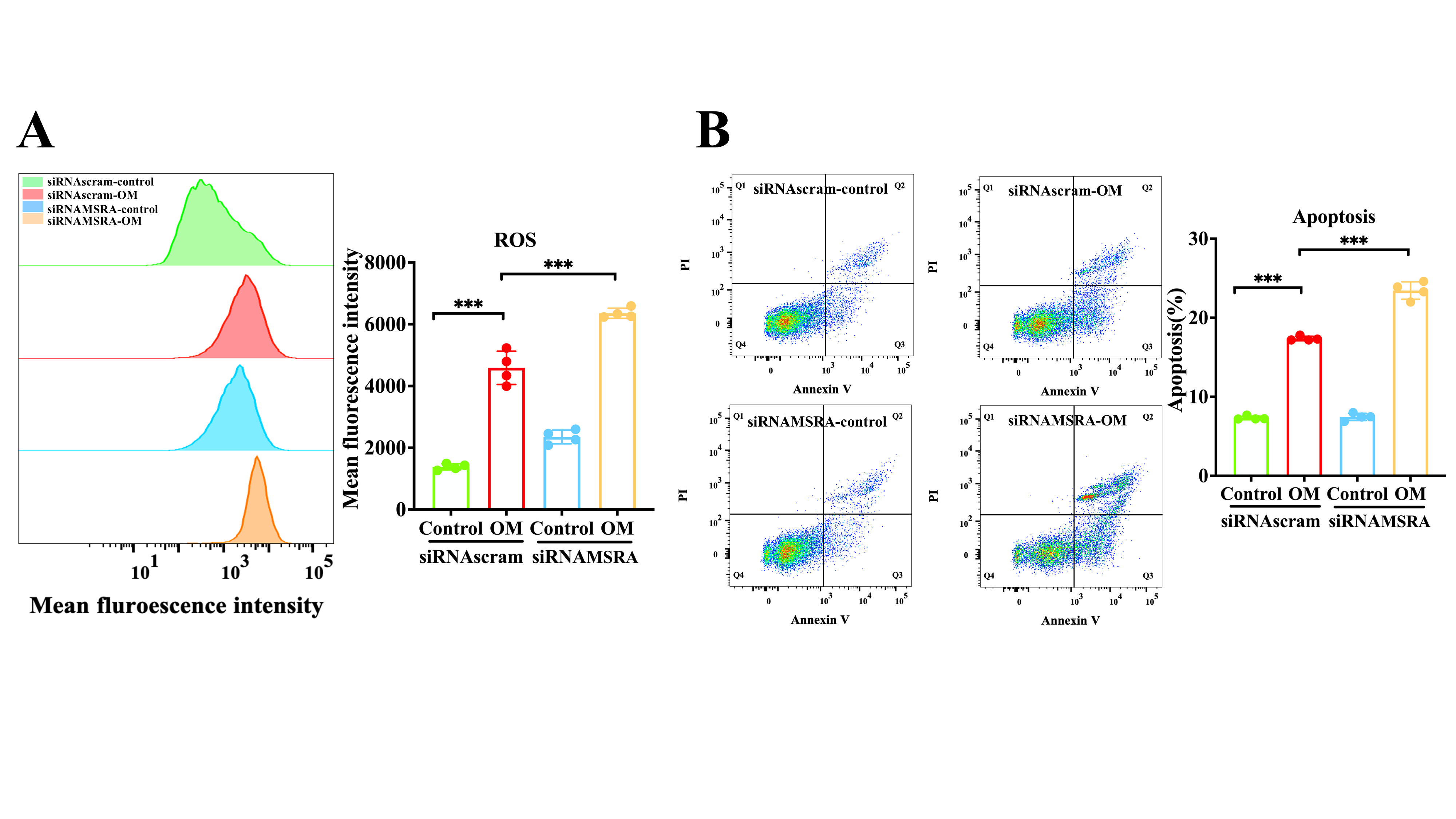


**Supplementary figure 3.** **Alkaline phosphatase staining for calcium deposition in VICs after MSRA overexpression (n=6 for each group), scale bar: 5mm and 500μm.** Data are presented as means ± SEM and compared by one-way analysis of variance followed by Bonferroni post-hoc test. VICs, valve interstitial cells; MSRA, methionine sulfoxide reductase A; OM, osteogenic medium.

**
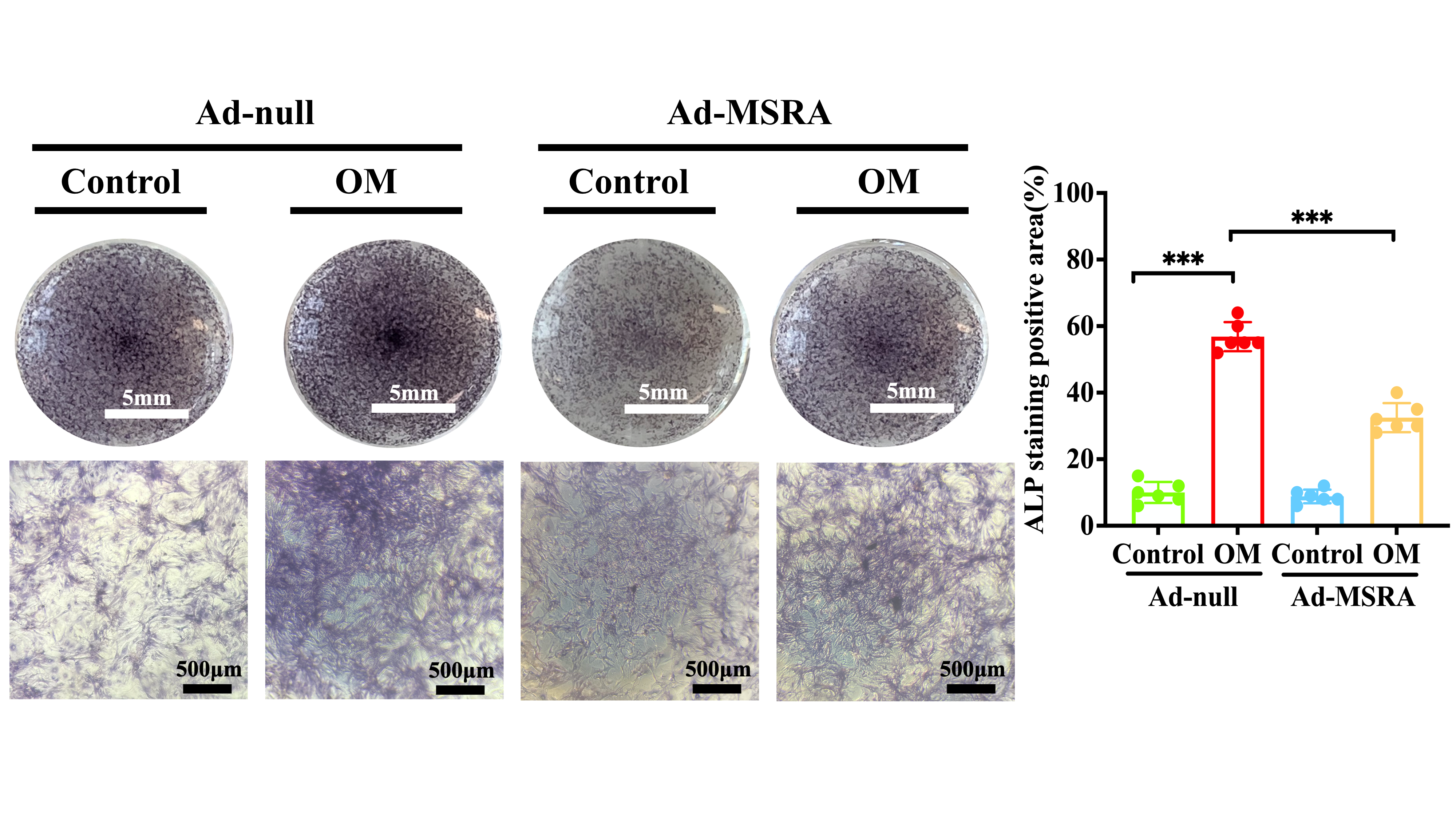
**

**Supplementary figure 4.** **MSRA overexpression reduces (A) ROS production and (B) apoptotic cell formation in VICs culture with osteogenic medium (n=4 for each group).** Data are presented as means ± SEM and compared by one-way analysis of variance followed by Bonferroni post-hoc test. MSRA, methionine sulfoxide reductase A; ROS, reactive oxygen species; VICs, valvular interstitial cells; OM, osteogenic medium.


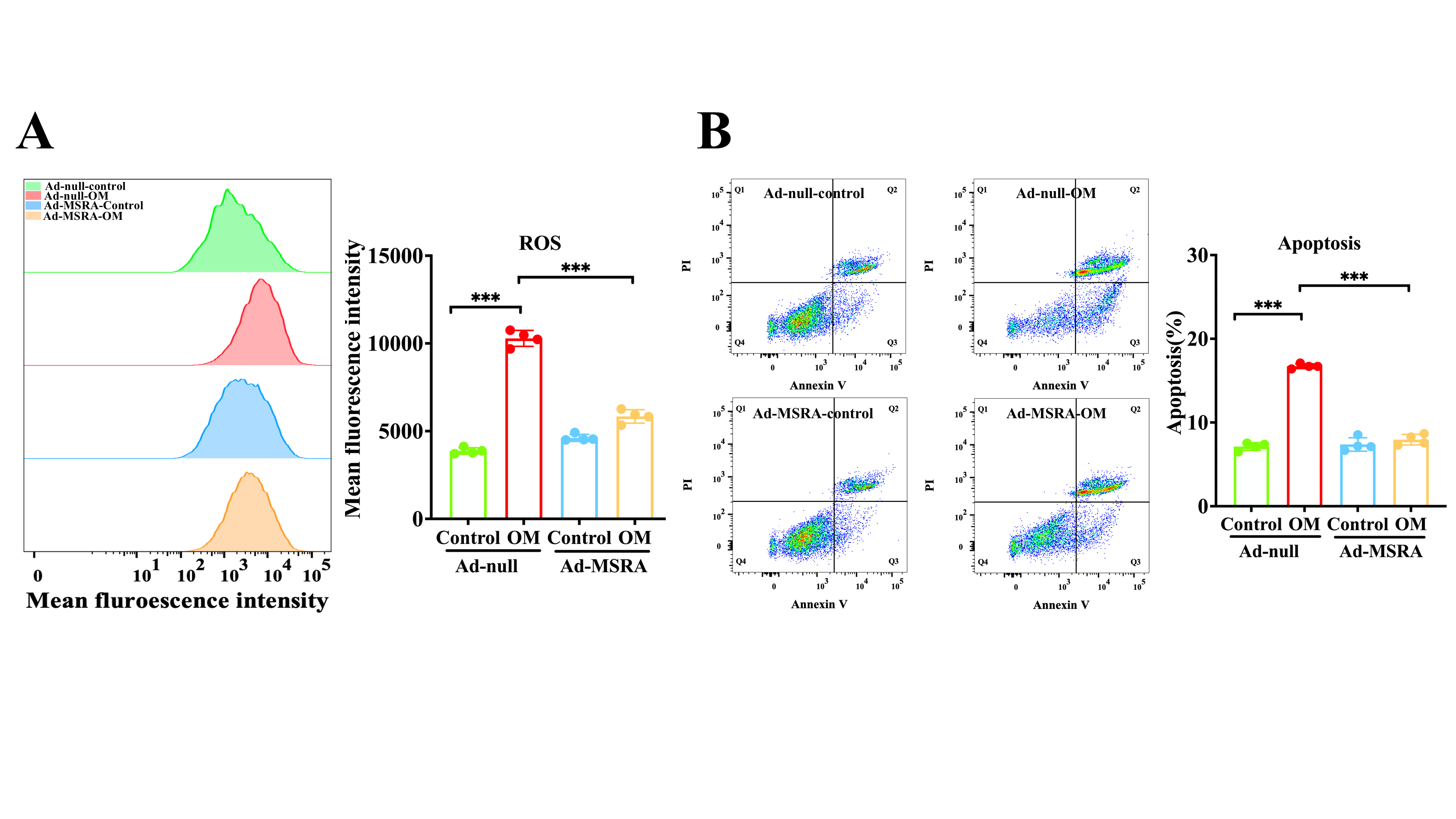


**Supplementary figure 5.** **MSRA expression is increased in senescent VICs. (A)** Western blot analysis and quantification of P21, OCN, RUNX2, OPN, and MSRA protein expression in aging VICs (n=6 for each group). **(B)** Immunofluorescence staining (n=6 for each group) and **(C)** flow cytometry (n=4 for each group) for P21(magenta), RUNX2(red) and MSRA (green) in aging VICs, scale bar: 20μm. Data are presented as mean ± SEM and compared by one-way analysis of variance followed by Bonferroni post-hoc test. MSRA, methionine sulfoxide reductase A; H_2_O_2_, hydrogen peroxide; P21, cyclin-dependent kinase inhibitor 1A; OCN, osteocalcin; RUNX2, runt-related transcription factor; OPN, osteopontin; VICs, valvular interstitial cells; DAPI, 4′,6-diamidino-2-phenylindole. NS, not significant; * *P* < 0.05.


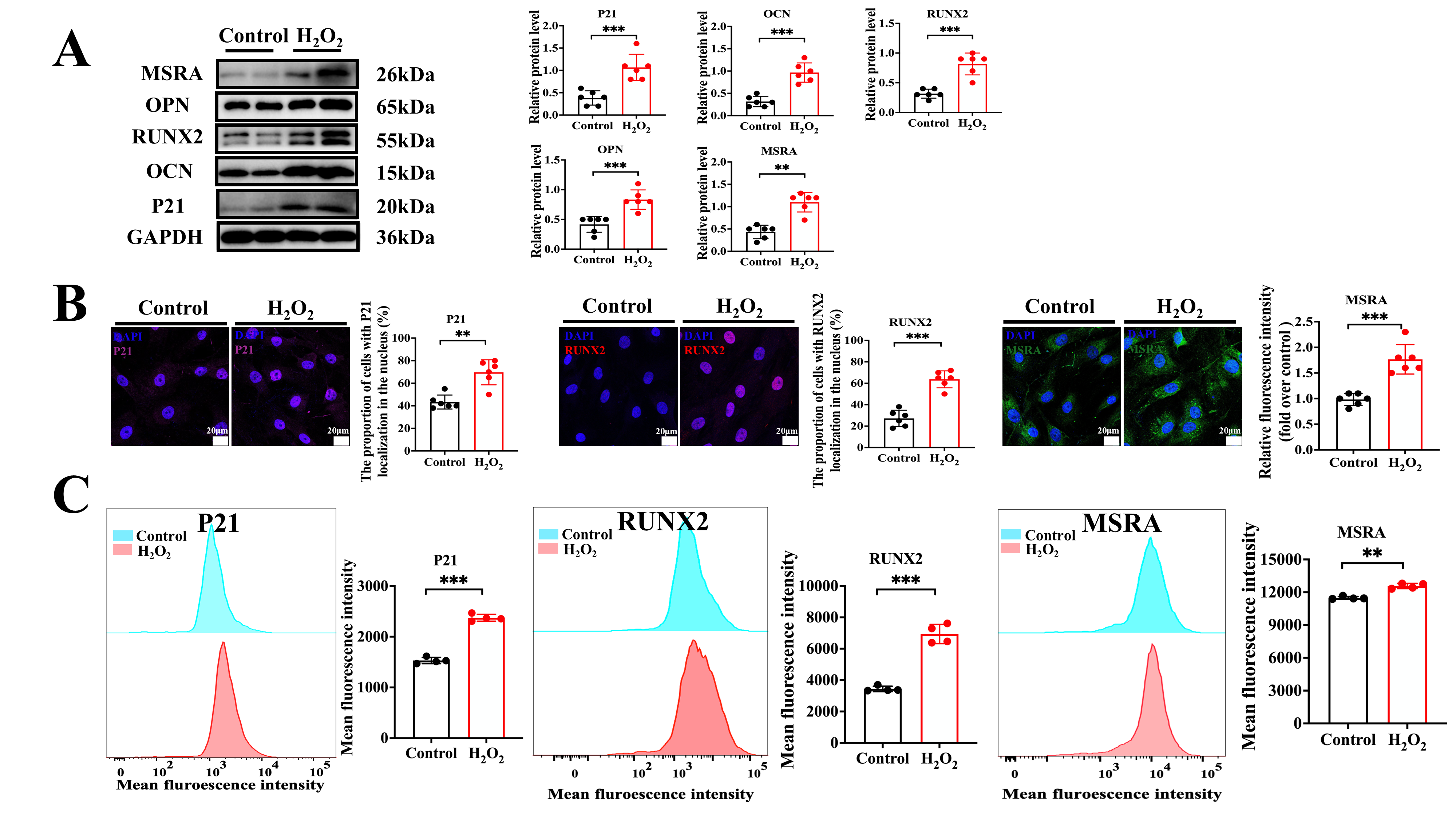


**Supplementary figure 6.** **Senescent stimulus H_2_O_2_ exacerbated OM-induced VIC calcification.** Western blot analysis and quantification of P21, OCN, RUNX2, and OPN protein expression in VICs (n=6 for each group). Data are presented as mean ± SEM and compared by one-way analysis of variance followed by Bonferroni post-hoc test. MSRA, methionine sulfoxide reductase A; H_2_O_2_, hydrogen peroxide; OM, osteogenic medium; P21, cyclin-dependent kinase inhibitor 1A; OCN, osteocalcin; RUNX2, runt-related transcription factor; OPN, osteopontin; VICs, valvular interstitial cells; DAPI, 4′,6-diamidino-2-phenylindole. NS, not significant; * *P* < 0.05.

**
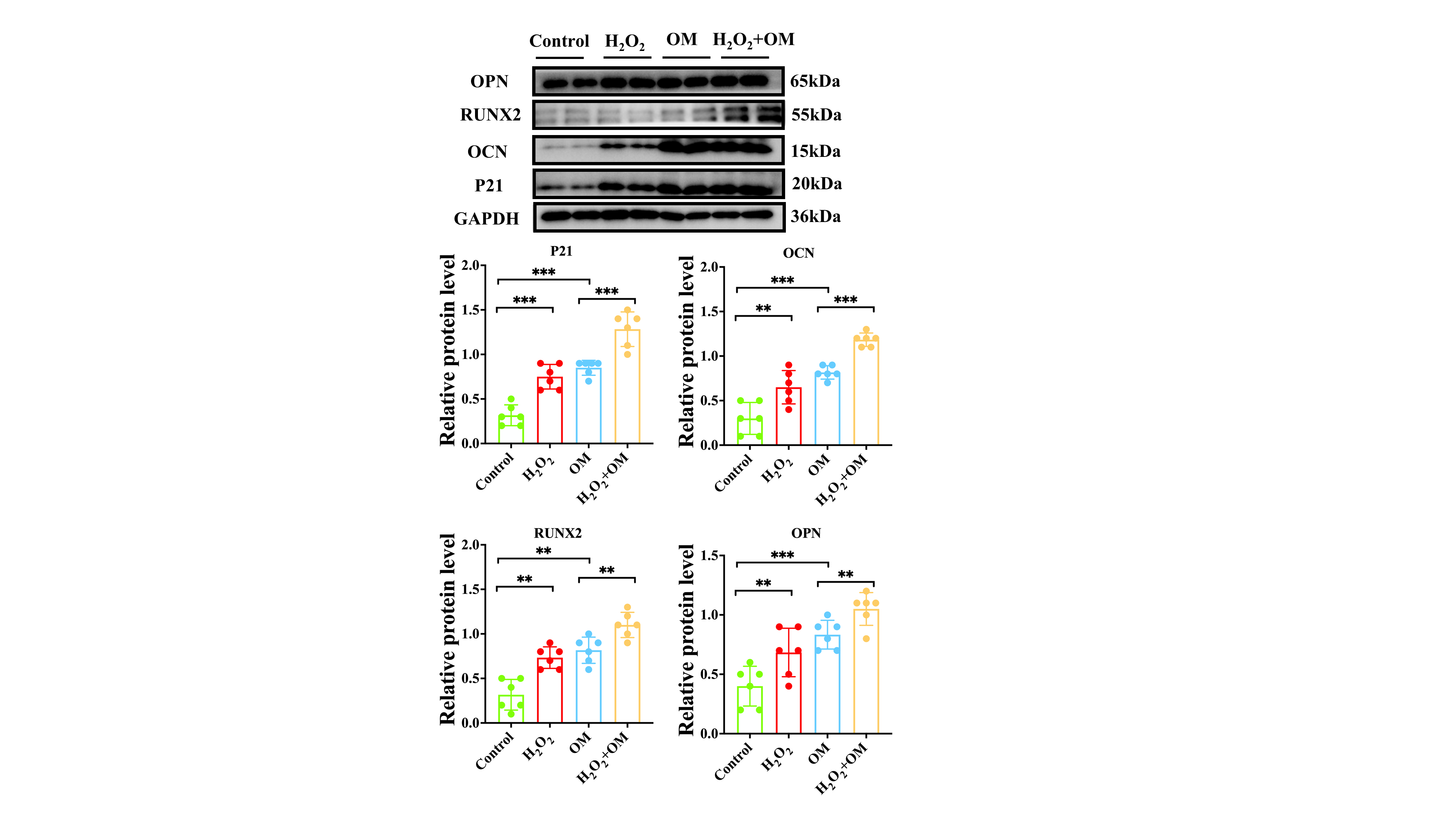
**

**Supplementary figure 7.** **MSRA silencing promotes VIC senescence *in vitro*.** Flow cytometry for **(A)** P21 and **(B)** RUNX2 in VICs under H_2_O_2_ stimulus after MSRA silencing (n=4 for each group). **(C)** SA-β-galactosidase staining for senescent VICs after MSRA silencing (n=6 for each group). Data are presented as means ± SEM compared by student’s t-test or one-way analysis of variance followed by Bonferroni post-hoc test. P21, cyclin-dependent kinase inhibitor 1A; RUNX2, runt-related transcription factor; VICs, valvular interstitial cells; H_2_O_2_, hydrogen peroxide; MSRA, methionine sulfoxide reductase A. NS, not significant; * *P* < 0.05, ***P* < 0.01, ****P* < 0.001.

**
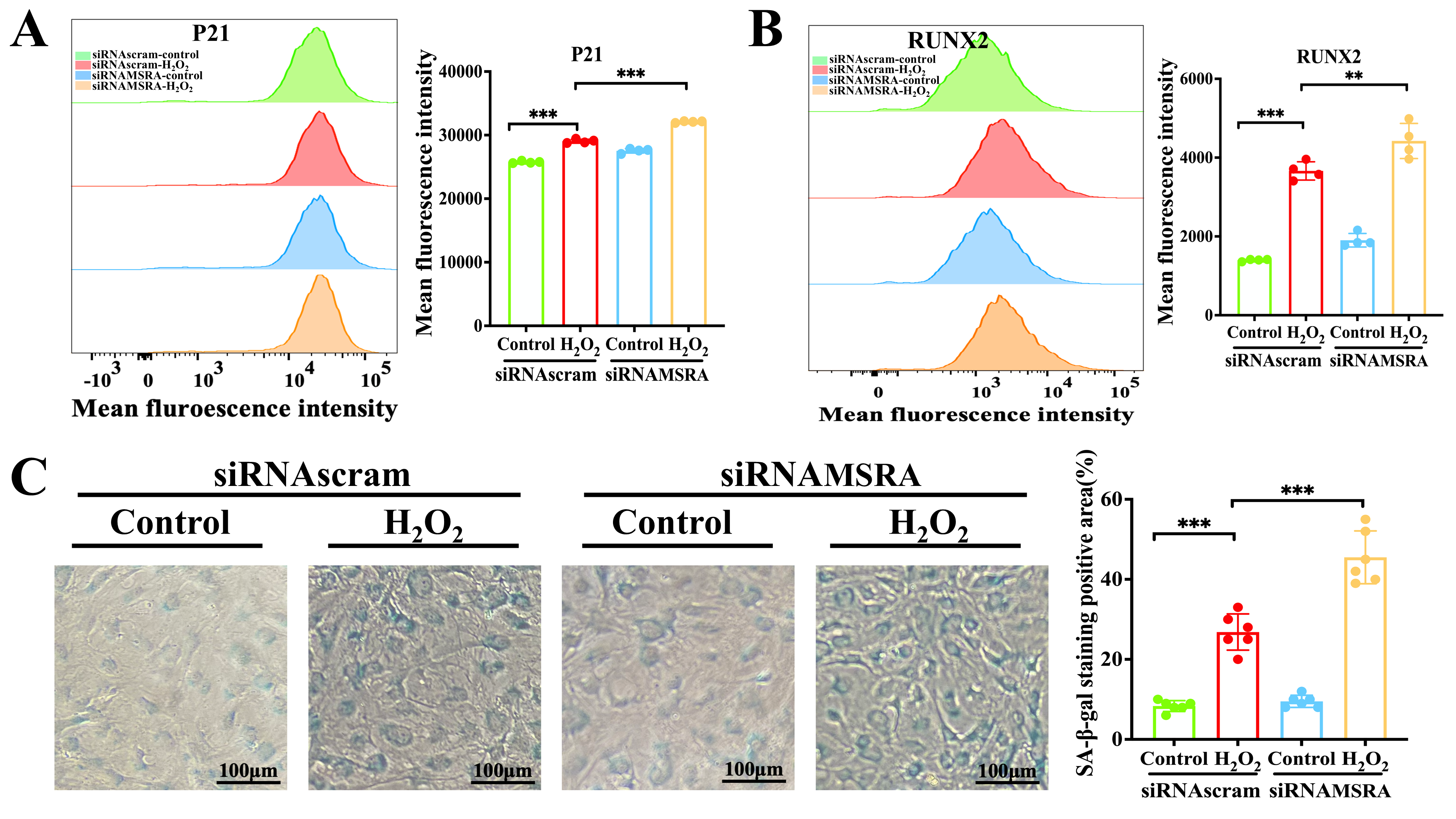
**

**Supplementary figure 8.** **MSRA overexpression alleviates VIC senescence *in vitro*.** Flow cytometry for **(A)** P21 and **(B)** RUNX2 in VICs under H_2_O_2_ stimulus after MSRA overexpression (n=4 for each group). **(C)** SA-β-galactosidase staining for senescent VICs after MSRA overexpression (n=6 for each group). Data are presented as means ± SEM and compared by student’s t-test or one-way analysis of variance followed by Bonferroni post-hoc test. P21, cyclin-dependent kinase inhibitor 1A; RUNX2, runt-related transcription factor; VICs, valvular interstitial cells; H_2_O_2_, hydrogen peroxide; MSRA, methionine sulfoxide reductase A. NS, not significant; * *P* < 0.05, ***P* < 0.01, ****P* < 0.001.

**
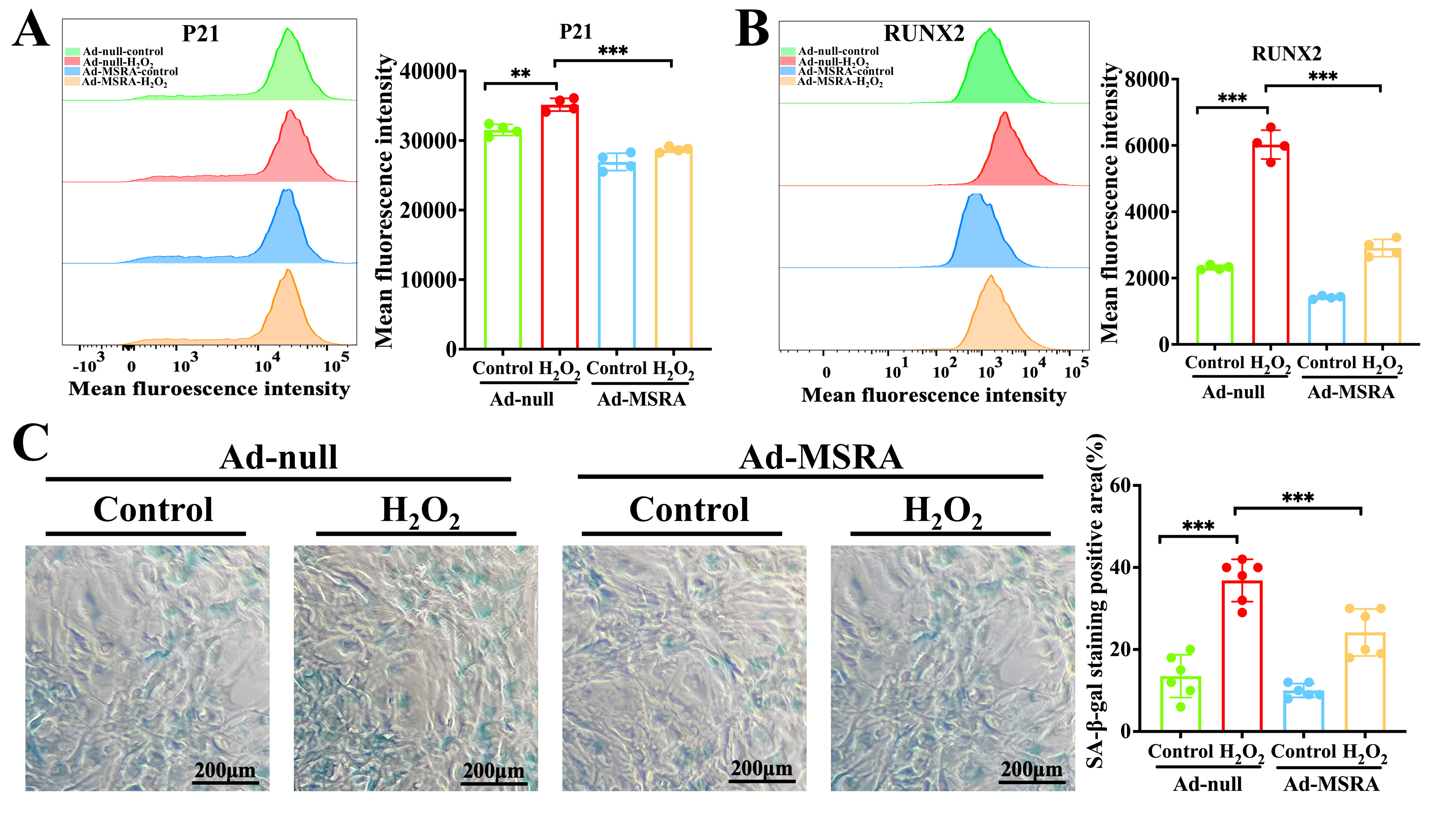
**

**Supplementary figure 9.** **MSRA silencing on TLRs family mRNA levels** (n=6 for each group)**.** Data are presented as mean ± SEM and compared by student’s t-test or one-way analysis of variance followed by Bonferroni post-hoc test. MSRA, methionine sulfoxide reductase A; TLR, toll-like receptor; OM, osteogenic medium. NS, not significant; * *P* < 0.05.


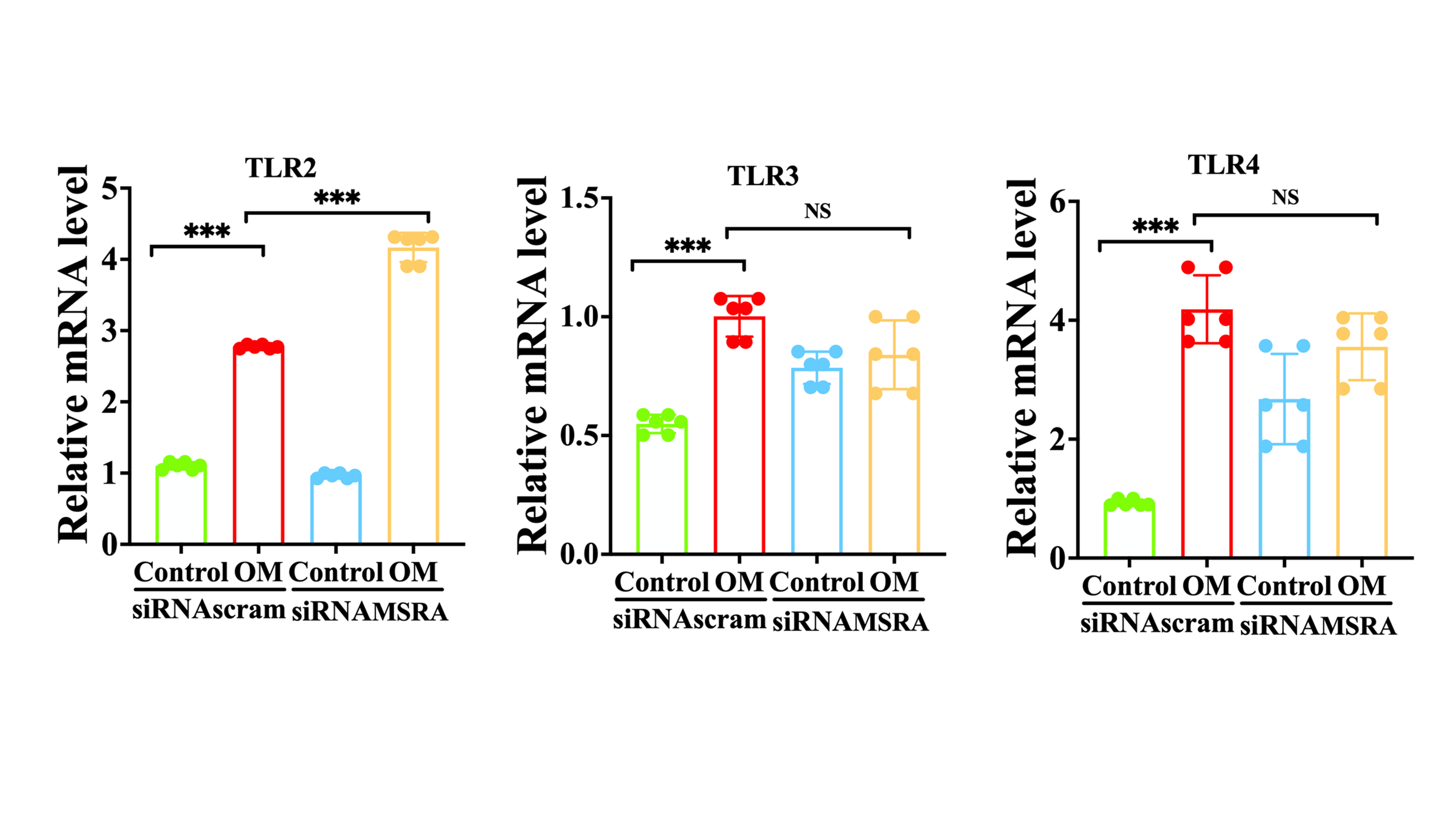


**Supplementary figure 10.** **MSRA was overexpressed in the aortic valve leaflets after administrating AAV2-MSRA**. (**A**) Quantitative real-time polymerase chain reaction analysis of MSRA mRNA expression levels in the heart of ApoE^-/-^ mice (n=9 for each ND group, n=10 for each HCD group). (**B**) Immunohistochemical staining showing MSRA expression in ApoE^-/-^ mice (n=9 for each ND group, n=10 for each HCD group), scale bar: 250μm. Data are presented as mean ± SEM and compared by student’s t-test or one-way analysis of variance followed by Bonferroni post-hoc test. MSRA, methionine sulfoxide reductase A; AAV2, adeno-associated virus subtype 2; ND, normal diet; HCD, high cholesterol diet. NS, not significant; * *P* < 0.05, ***P* < 0.01, ****P* < 0.001.


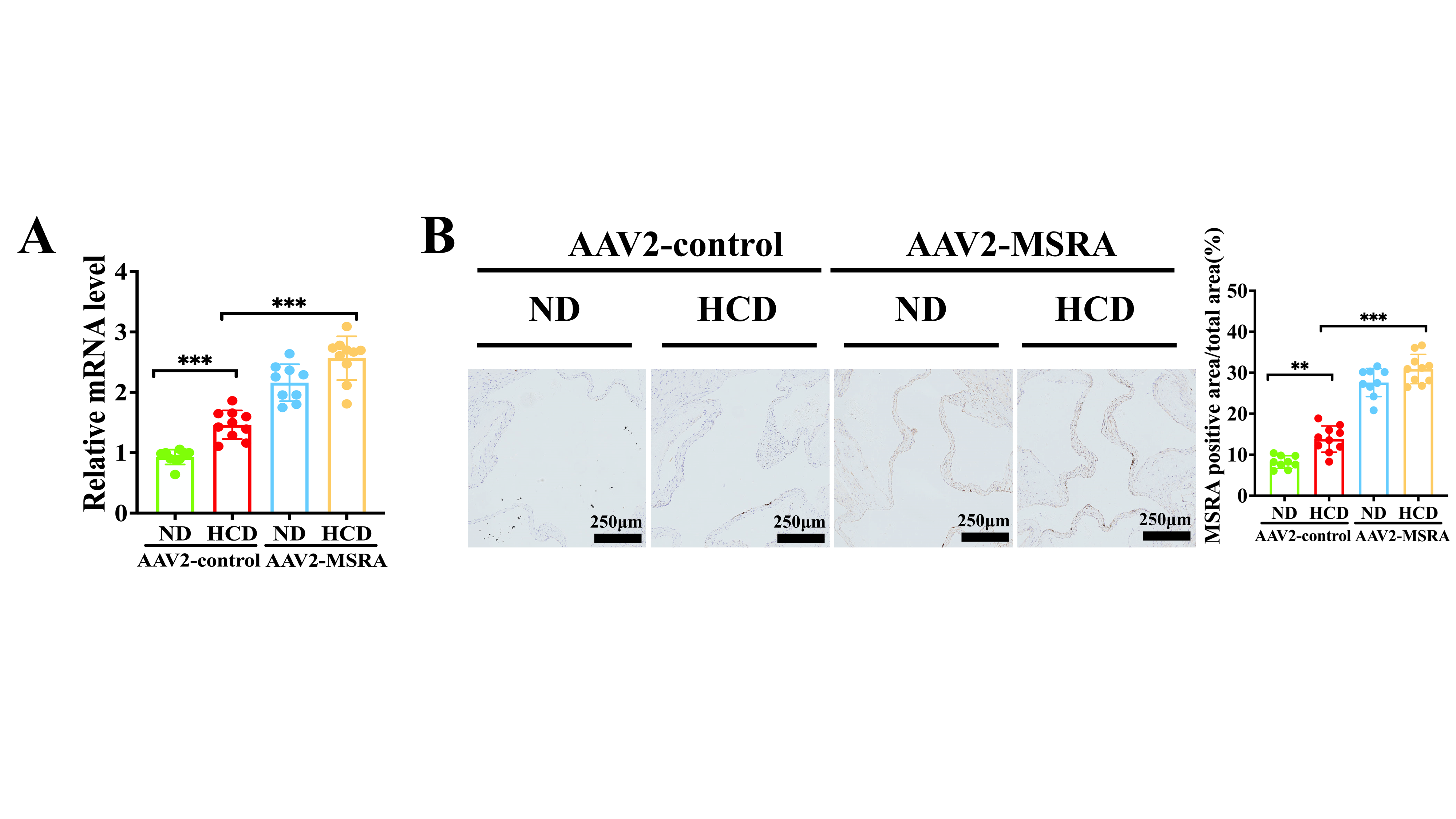


**Supplementary figure 11. MSRA is upregulated in the late stages of senescence. (A)** Quantitative real-time polymerase chain reaction analysis of MSRA mRNA expression levels in different stages of H_2_O_2_-induced senescence (n=6 for each group). **(B)** Western blot analysis and quantification of MSRA, P21, and RUNX2 protein expression in different stages of H_2_O_2_-induced senescence (n=6 for each group). **(C)** Flow cytometry analysis and the mean fluorescence intensity of reactive oxygen species in different stages of H_2_O_2_-induced senescence (n=4 for each group). Data are presented as means ± SEM and compared by student’s t-test or one-way analysis of variance followed by Bonferroni post-hoc test. MSRA, methionine sulfoxide reductase A; H_2_O_2_, hydrogen peroxide; P21, cyclin-dependent kinase inhibitor 1A; RUNX2, runt-related transcription factor; ROS, reactive oxygen species. NS, not significant; * *P* < 0.05, ***P* < 0.01, ****P* < 0.001.

**
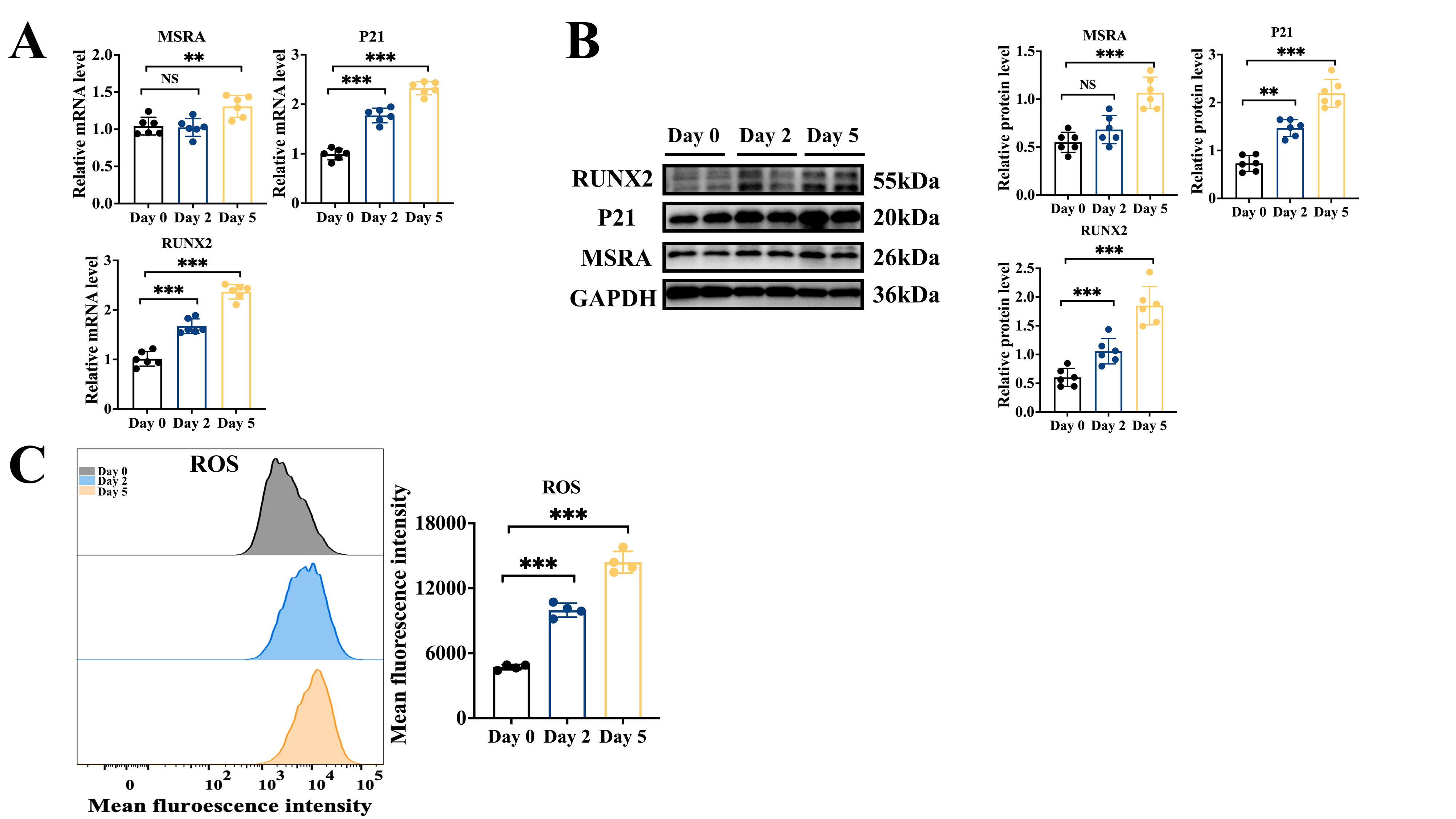
**
